# A Review of Formulations, Boundary Value Problems and Solutions for Numerical Computation of Transcranial Magnetic Stimulation Fields

**DOI:** 10.3390/brainsci13081142

**Published:** 2023-07-29

**Authors:** J. A. Pérez-Benítez, P. Martínez-Ortiz, J. Aguila-Muñoz

**Affiliations:** 1 Laboratorio de Bio-Electromagnetismo, ESIME-SEPI, Edif. Z-4, Instituto Politécnico Nacional, Mexico City 07738, CDMX, Mexico; pmartinezo@ipn.mx; 2 CONAHCYT—Centro de Nanociencias y Nanotecnología, Universidad Nacional Autónoma de México, km 107 Carretera Tijuana-Ensenada, Apartado Postal 14, Ensenada 22800, BC, Mexico

**Keywords:** transcranial magnetic stimulation, review, formulations, boundary value problems, review of numerical methods

## Abstract

Since the inception of the transcranial magnetic stimulation (TMS) technique, it has become imperative to numerically compute the distribution of the electric field induced in the brain. Various models of the coil-brain system have been proposed for this purpose. These models yield a set of formulations and boundary conditions that can be employed to calculate the induced electric field. However, the literature on TMS simulation presents several of these formulations, leading to potential confusion regarding the interpretation and contribution of each source of electric field. The present study undertakes an extensive compilation of widely utilized formulations, boundary value problems and numerical solutions employed in TMS fields simulations, analyzing the advantages and disadvantages associated with each used formulation and numerical method. Additionally, it explores the implementation strategies employed for their numerical computation. Furthermore, this work provides numerical expressions that can be utilized for the numerical computation of TMS fields using the finite difference and finite element methods. Notably, some of these expressions are deduced within the present study. Finally, an overview of some of the most significant results obtained from numerical computation of TMS fields is presented. The aim of this work is to serve as a guide for future research endeavors concerning the numerical simulation of TMS.

## 1. Introduction

Transcranial magnetic stimulation (TMS) is a method that modulates brain activity using an electric field induced in the brain by potent magnetic field pulses [1,2,3,4]. This method is based on the application of magnetic field pulses to the brain cortex using coils, inducing electric fields oriented along certain paths to stimulate or inhibit brain activity in these areas. Several works [5,6,7,8,9] have shown that after TMS treatments, patients show improvement in their conditions, such as depression [5], mania, obsessive–compulsive disorder, post-traumatic stress disorder and schizophrenia [6,7,8,9]. TMS in combination with EEG methods [10,11] can also be used as a tool too study brain functions. However, the efficacy of TMS for treatments is highly influenced by factors such as the efficiency [12,13], focality [14,15,16,17,18,19] and convenient distribution [15] of the induced electric fields. Furthermore, these factors are determined by several parameters, such as the quantity, [16,20], arrangement [16,17,20] and geometric parameters of excitation coils [16,18,21,22]; coil distance and orientation with respect to the brain [20,23,24]; excitation current waveform [25,26,27,28,29]; and electric properties of the brain tissues [30,31,32,33,34], among others.

Based on the studies of the influence of the aforementioned parameters on TMS, previous works [35,36,37,38,39,40,41] have analyzed the general mechanisms associated with TMS. From these works, it can be deduced that to optimize the excitation parameter for each TMS application, avoiding ethical conflicts, and for a practical and cost-effective estimation of the effect of the excitation electric field on neuronal activity, it is necessary to use simulation methods. In particular, Roth and Basser [42] proposed a method for computing the electric field and its gradient along a nerve induced by a single coil. They modeled the nerve as a passive cable whose electric potential is influenced by an excitatory electric field gradient. The excitatory electric field corresponds to the primary electric field [42,43,44], which depends only on the magnetic potential vector rate [45,46], without considering the effect of the scalar potential. This approximation was used in the some of initial TMS simulation works [42,43,44] and os still widely used but for coil parameter optimization [21,22]. However, it was later demonstrated [47,48,49,50,51] that a more precise estimation of the actual electric field induced in the brain tissues requires consideration of the secondary electric field equal to the negative gradient of the scalar potential. The scalar potential results from the electric charges formed at the tissue interfaces [47,48,49,50,51]. From this point, most of the works dedicated to the computation of the induced electric field [38,52,53,54,55,56,57] have proposed increasingly more realistic models for the coils, brain geometry and tissue properties and different formulations based on quasistatic approximation of the Maxwell equations [45,46]. In particular, quasistatic approximations have varied from magnetic quasi-static Laplace to magnetic quasistatic Poisson formulations, and with improvement of finite-element software, it was possible to include most of the source and potential contributions. However, the simultaneous use of complex coils, realistic brain geometry and complex formulations does not come for free, and even with the use of advanced software, the computational time increases substantially. This fact has brought forward questions such as: how much detail of the coil geometry is necessary to estimate the actual electric field induced by the real coil [58]? The development of new numerical methods such as neural networks [59] has also been motivated in order to increase the computation speed of TMS fields. Nevertheless, the most common approach has been the use of specific formulations of quasistatic approximation of Maxwell equations depending on the TMS applications, which is likely to remain the dominant approach for future TMS numerical computation, which, due to the microscopic size of neurons and nerves, requires increasing resolution and precision. Therefore, it is necessary to know the limitations and advantages of different formulations, as well as the different available numerical methods for their solutions.

## 2. Scope and Contributions

The TMS formulations analyzed in the present work correspond to the Maxwell equation potential representations and their quasistatic approximations, which are solved using numerical methods [1,2,3,4,5,6,7,8,9,14,15,16,17,18,19,20,21,22,23,24,25,26,27,28,29,30,31,32,33,34,35,36,37,38,39,40,41,42,43,44,45,46,47,48,49,50,51,52,53,54,55,56,57,60,61,62,63,64,65,66,67,68,69,70,71,72,73,74,75,76,77,78,79,80,81,82,83,84,85,86,87,88,89,90,91,92,93,94,95,96,97,98]. Other methods such as the impedance method [99] and neural network methods [59] are not considered in this review. The methods that start from Maxwell equations but derive non-differential equation representations such as dipole-based methods [100] are also not considered in the present analysis.

The objective of this study is to serve as a comprehensive guide for the numerical computation of TMS fields by reviewing previous works in this field. To achieve this, a series of deductions leading to various quasistatic approximations for the computation of TMS fields are presented, followed by a review of works that have utilized these approximations. Furthermore, the numerical solutions using finite difference and finite element methods are discussed in detail, drawing upon existing methods proposed in the literature. Additionally, this study deduces methods not found in the consulted literature, such as finite difference for quasistatic magnetic A-ϕ and Darwin models and finite element for quasistatic magnetic A-ϕ and Darwin models using the Galerkin method, as well as their implementation of boundary conditions applied to TMS fields.

The papers included in this review pertaining to field simulations in TMS were systematically chosen in a chronological manner, with emphasis placed on commencing with highly impactful publications based on their citation count. Additionally, works that cited these influential papers while introducing novel variants of formulations and numerical methods were also considered. It is essential to acknowledge that not all the works encompassing TMS field simulations propose new formulations or numerical methods. In fact, a significant portion of the TMS field simulation literature references the original works that introduced these methods, with their contributions primarily concentrated in the domain of applications. Every effort was made to incorporate as many of these application-oriented works as feasible.

## 3. Field Theory of TMS

### 3.1. Maxwell Equations and Their Representation Using Vector and Scalar Potentials

To reduce the computational time and facilitate the numerical computation of TMS fields, some quasistatic approximations of the Maxwell equations [45,46] or, more specifically, their vector and scalar representation have been proposed [42,43,44,47,48,49,50,51]. Each of the formulations described below, some of which were used in previous TMS works, is derived from the potential representation of Maxwell equations. The classical forms of Maxwell equations are the Maxwell–Faraday, Maxwell–Ampere, Maxwell–Thompson and Maxwell–Gauss equations, expressed, in that order, as follows:

(1a) $$\begin{array}{cc} \; & \vec{\nabla }\times \vec{E}=-\frac{\partial \vec{B}}{\partial t} \end{array}$$

(1b) $$\begin{array}{cc} \; & \vec{\nabla }\times \vec{H}=\vec{J_{s}}+{\vec{J}}_{ind}+\frac{\partial \vec{D}}{\partial t} \end{array}$$

(1c) $$\begin{array}{cc} \; & \vec{\nabla }\;\cdot \;\vec{B}=0 \end{array}$$

(1d) $$\begin{array}{cc} \; & \vec{\nabla }\;\cdot \;\vec{D}=\rho \end{array}$$

The constitutive equations should also be considered:

(2a) $$\begin{array}{cc} \; & \vec{D}=\epsilon \vec{E}=\epsilon_{o}\epsilon_{r}\vec{E} \end{array}$$

(2b) $$\begin{array}{cc} \; & \vec{B}=\mu \vec{H}=\mu_{o}\mu_{r}\vec{H}, \end{array}$$

as well as Ohm’s law

(3) $$\vec{J}=\sigma \vec{E}$$

which establish the relation between the current density and the electric field. The Maxwell equations could be expressed using vector and scalar potentials [45,46]. Since the divergence of the curl of a vector is always zero, $\vec{\nabla }\;\cdot \;\left(\vec{\nabla }\times \vec{A}\right)=0$. Then, *B*, the divergence of which is zero according to the Maxwell–Thompson equation ($\vec{\nabla }\;\cdot \;\vec{B}=0$), could be replaced by the curl of an arbitrary vector such that:

(4) $$\vec{B}=\vec{\nabla }\times \vec{A}$$

where $\vec{A}$ is the vector magnetic potential. Substituting this expression into the Maxwell–Faraday’s Equation (1a) and considering the distributive and commutative properties of the curl and the partial derivative, the following expression is obtained:

(5) $$\vec{\nabla }\times \left(\vec{E}+\frac{\partial \vec{A}}{\partial t}\right)=0$$

According to Jackson [45], $\vec{\nabla }\times \left(-\vec{\nabla }\phi \right)=0$ for any scalar function as it is, for example, for the electric scalar potential (ϕ). Then, the term inside the curl can be expressed as $\vec{E}+\frac{\partial \vec{A}}{\partial t}=-\vec{\nabla }\phi$ and resolving with respect to $\vec{E}$:

(6) $$\vec{E}=-\frac{\partial \vec{A}}{\partial t}-\vec{\nabla }\phi$$

Substituting Equations (3) and (6) and the constitutive Equation (2) into the Maxwell–Ampere Equation (1b) results in:

(7) $$\vec{\nabla }\times \left(\frac{1}{\mu }\vec{\nabla }\times \vec{A}\right)+\sigma \left(\frac{\partial A}{\partial t}+\nabla \phi \right)+\frac{\partial }{\partial t}\left(\epsilon \left(\frac{\partial A}{\partial t}+\nabla \phi \right)\right)={\vec{J}}_{s}$$

Applying the divergence operator to this equation and taking into account the aforementioned identity, i.e., that the divergence of the curl of a vector is always zero, gives:

(8) $$\vec{\nabla }\;\cdot \;\left(\sigma \left(\frac{\partial \vec{A}}{\partial t}+\vec{\nabla }\phi \right)\right)+\vec{\nabla }\;\cdot \;\frac{\partial }{\partial t}\left(\epsilon \left(\frac{\partial \vec{A}}{\partial t}+\vec{\nabla }\phi \right)\right)=\vec{\nabla }\;\cdot \;J_{s}$$

where ${\vec{J}}_{s}$ is the external current density.

Using Equations (7) and (8), the full Maxwell equations are represented using vector and scalar potentials. However, the computational cost required to solve this set of differential equations could be significantly high depending on the geometry of the domains and their electric and magnetic properties. These equations could be simplified by making some considerations valid for TMS excitation characteristics and electric and magnetic properties of brain tissue. The formulations corresponding to this approximations are referred to in the literature as quasistatic approximations. The most well-known quasistatic approximations are [45,60,61] electro-quasistatic (EQS) approximation, which enables modeling of the potentials under the influence of conduction and displacement currents, including capacitive-resistive effects, but neglects the inductive phenomena; magnetic quasistatic (MQS) approximation, which neglects displacement current and capacitive effects and considers inductive phenomena; and the Darwin model, which comprises short-off electromagnetic quasistatic approximation and allows for the inclusion of inductive phenomena, as well as capacitive effects.

### 3.2. Poisson MQS ϕ-Formulation

The principle of TMS stimulation is that an electric field is induced in the brain by a time-varying magnetic field. The magnetic field is produced by a coil (or coils) fed by current peaks. Therefore, it is impossible to neglect inductive phenomena. Consequently, the behavior of potential can be computed using MQS. In MQS, the most restrictive formulation is the MQS-ϕ formulation, which is based on three approximations:

1. The wavelength of the excitation field is significantly higher than the size of the head. For TMS pulses with a duration of τ≥0.1 ms corresponding to a frequency of f≤10 kHz, the corresponding wavelength is λ≥300 m, which is much higher than the head dimensions;
2. Diminishment of the capacitive effects in the brain tissue: Resulting from continuity conditions of electric currents in the interface between materials with different electric conductivities [45], the electric charges accumulate in this area, which could provoke capacitive effects. However, under this quasistatic approximation, induced charges are considered to move freely inside the brain, not allowing for static accumulation of charges that could generate capacitive effects. Furthermore, polarization effects are not considered;
3. Neglecting the skin-effect: A time-varying magnetic field induces electric currents that oppose the magnetic field. The amplitude of the induced current is proportional to the electric conductivity. Consequently, when a magnetic field enters a medium with an electric conductivity other than zero, it decays as it penetrates in the medium. However, the electric conductivity of brain tissue is low ($\sigma_{whitematter}=0.15$ S/m), and together with the paramagnetic magnetic properties of these tissues, $\delta \;=\;1/\sqrt{\pi f\mu \sigma }\approx 5$ m [62], which confirms this approximation.

To apply these approximations to Equations (7) and (8), it is important to explain the cause–effect relations in these equations. According to Equation (6), Ohm’s Law and the constitutive equation for the electric flux density, Equation (7) can be expressed as:

(9) $$\vec{\nabla }\times \left(\frac{1}{\mu }\vec{\nabla }\times \vec{A}\right)={\vec{J}}_{s}+{\vec{J}}_{ind}+{\vec{J}}_{disp}$$

where $J_{ind}=\sigma \vec{E}=-\sigma \left(\frac{\partial A}{\partial t}+\nabla \phi \right)$ are the induced conductive currents or eddy currents, and $J_{disp}=\frac{\partial \vec{D}}{\partial t}=\frac{\partial }{\partial t}\left(\epsilon \vec{E}\right)=-\frac{\partial }{\partial }\left(\epsilon \left(\frac{\partial A}{\partial t}+\nabla \phi \right)\right)$ are the displacement currents. This expression shows that the magnetic field is induced by an external current whose value decreases due to the induced currents. The induced currents can be computed according to Equation (8), which can be rewritten as:

(10) $$\vec{\nabla }\;\cdot \;{\vec{J}}_{ind}+\vec{\nabla }\;\cdot \;{\vec{J}}_{disp}=-\vec{\nabla }\;\cdot \;{\vec{J}}_{s}$$

The simplification of the MQS ϕ-formulation implies neglect of the effects of eddy currents on the magnetic field (no skin effect) and of the displacement currents (no capacitive effects). Consequently, this approximation implies removing $J_{ind}$ and $J_{dis}$ from Equation (9). This approximation does not mean that the induced current is zero but that $J_{ind}<<J_{s}$. Furthermore, all the materials involved in the phenomenon are paramagnetic ($\mu_{r}=1$). Therefore, Equation (9) becomes:

(11) $$\vec{\nabla }\times \left(\vec{\nabla }\times \vec{A}\right)=\mu_{0}{\vec{J}}_{s}$$

Since the vector magnetic potential is not unique, a restriction such as the Coulomb gauge ($\nabla \;\cdot \;\vec{A}=0$) could be used [45], which, together with the identity $\vec{\nabla }\times \vec{\nabla }\times \vec{A}=\vec{\nabla }\left(\vec{\nabla }\;\cdot \;\vec{A}\right)-\nabla^{2}\vec{A}$, modifies Equation (9) as follows:

(12) $$\nabla^{2}\vec{A}=-\mu_{0}{\vec{J}}_{s}$$

On the other hand, in the domain of the brain, neglecting the displacement current, $J_{dis}=0$, and since there is no external current, ${\vec{J}}_{s}=0$, and Equation (10) becomes:

(13) $$\vec{\nabla }\;\cdot \;{\vec{J}}_{ind}=0$$

or

(14) $$\vec{\nabla }\;\cdot \;\left(\sigma \left(\frac{\partial \vec{A}}{\partial t}+\vec{\nabla }\phi \right)\right)=0$$

which could also be expressed as:

(15) $$\vec{\nabla }\;\cdot \;\left(\sigma \vec{\nabla }\phi \right)=-\vec{\nabla }\;\cdot \;\left(\sigma \frac{\partial \vec{A}}{\partial t}\right)$$

Expressions (12) and (15) could be used to obtain $\vec{A}\left(r,t\right)$ and ϕ(r,t), which allow for computation of the TMS-induced electric field ($\vec{E}$) using Equation (6). For this purpose, the electric field is divided in two components: $\vec{E}={\vec{E}}_{p}+{\vec{E}}_{s}$, where:

(16) $$E_{p}=-\frac{\partial \vec{A}}{\partial t}$$

is called the primary electric field and

(17) $$E_{s}=-\nabla \phi$$

is the secondary electric field.

This formulation can be called the Poisson MQS ϕ-formulation, since both expressions are Poisson equations. This is also a ϕ formulation because $\vec{A}$ and ϕ are only partially coupled. The scalar electric potential (ϕ) is produced by electric charges induced by the time variation of the vector magnetic potential ($\vec{A}$), but the influence of the electric field on the magnetic potential is neglected. This approximation has the advantage that it could be easily solved using numerical methods with low computational resources. However, it is necessary to consider the aforementioned limitations of the model.

### 3.3. Laplace MQS ϕ Formulation

A further simplification of the Poisson MQS formulation consists of considering that the electric charges responsible for the secondary electric field are formed only at the brain-to-air interface. The principle of TMS stimulation is that an electric field is induced in the brain by a time-varying magnetic field. The magnetic field is produced by a coil (or coils) fed by current peaks. The accumulation of charges at the brain-to-air interface could be explained by considering the following principles: The primary electric field ($E_{p}$) is computed as if the medium were isotropic and uniform. Therefore, $E_{p}$ is continuous across all interfaces.

(18) $$\vec{E_{p}^{1}}\;\cdot \;\vec{n}=\vec{E_{p}^{2}}\;\cdot \;\vec{n}=\vec{E_{p}}\;\cdot \;\vec{n}$$

According Ohm’s Law, $\vec{J_{i}^{p}}=\sigma_{i}\vec{E_{p}}$, and considering that electric conductivities of the interfacing areas are different, ($\sigma_{1}\neq \sigma_{2}$), at the interfaces:

(19) $$\sigma_{1}\vec{E_{p}}\;\cdot \;\vec{n}\neq \sigma_{2}\vec{E_{p}}\;\cdot \;\vec{n}$$

Therefore,

(20) $$\vec{J_{p}^{1}}\;\cdot \;\vec{n}\neq \vec{J_{p}^{2}}\;\cdot \;\vec{n}$$

In the quasistatic limit, the normal component of total current crossing an interface between tissues is continuous, and it is given by the continuity law [45]:

(21) $$\vec{J^{1}}\;\cdot \;\vec{n}=\vec{J^{2}}\;\cdot \;\vec{n}$$

which seems to contradict (20). However, this law is satisfied by the formation of electric charges, which generate a secondary electric field ($\vec{E_{s}}$) and the corresponding current ($\vec{J_{s}^{i}}=\sigma_{i}\vec{E_{s}^{i}}$). In this case, Equation (21) can be expressed as:

(22) $$\left(\vec{J_{p}^{1}}+\vec{J_{s}^{1}}\right)\;\cdot \;\vec{n}=\left(\vec{J_{p}^{2}}+\vec{J_{s}^{2}}\right)\;\cdot \;\vec{n}$$

or

(23) $$\sigma_{1}\left(\vec{E_{p}}+\vec{E_{s}^{1}}\right)\;\cdot \;\vec{n}=\sigma_{2}\left(\vec{E_{p}}+\vec{E_{s}^{2}}\right)\;\cdot \;\vec{n}$$

The fact that the secondary fields in the media of the interface are different compensates for the total electric field in such a way that the continuity condition holds. In the case of the brain-to-air interface, $\sigma_{air}=\sigma_{2}=0$. Then, Equation (23) reduces to:

(24) $$\sigma_{1}\left(\vec{E_{p}}+\vec{E_{s}^{1}}\right)\;\cdot \;\vec{n}=0$$

or

(25) $$\left(\sigma_{1}\vec{\nabla }\phi \right)\;\cdot \;\vec{n}+\left(\sigma_{1}\frac{\partial \vec{A}}{\partial t}\right)\;\cdot \;\vec{n}=0$$

Since the charges and the corresponding electric currents are restricted to the brain boundary, which corresponds to the case when the brain model is made of a uniform tissue, then σ is considered to be uniform and isotropic inside the brain, and Equation (15) for the brain domain becomes:

(26) $${\vec{\nabla }}^{2}\phi =0$$

This Laplace equation can be used to compute the electric potential inside the brain domain. To obtain a unique and non-trivial solution from this equation, the Neumann boundary condition expressed by Equation (25) should be used. After obtaining ϕ from Equation (26) with the boundary condition (25) and $\vec{A}$ from Equation (12), the electric field is computed using Equation (6).

### 3.4. MQS A-ϕ Formulation

If only capacitive effects are neglected (${\vec{J}}_{dis}=0$) using the general transformations and considerations described in Section 3.2, Equations (7) and (8) can be expressed as:

(27a) $$\begin{array}{cc} \; & \sigma \left(\frac{\partial \vec{A}}{\partial t}+\nabla \phi \right)+\frac{1}{\mu_{0}}\vec{\nabla }\times \vec{\nabla }\times \vec{A}={\vec{J}}_{s} \end{array}$$

(27b) $$\begin{array}{cc} \; & \vec{\nabla }\;\cdot \;\left(\sigma \vec{\nabla }\phi \right)+\vec{\nabla }\;\cdot \;\left(\sigma \frac{\partial \vec{A}}{\partial t}\right)=0 \end{array}$$

or using the Coulomb gauge:

(28a) $$\begin{array}{cc} \; & \sigma \left(\frac{\partial \vec{A}}{\partial t}+\nabla \phi \right)-\frac{1}{\mu_{0}}\nabla^{2}\vec{A}={\vec{J}}_{s} \end{array}$$

(28b) $$\begin{array}{cc} \; & \vec{\nabla }\;\cdot \;\left(\sigma \vec{\nabla }\phi \right)+\vec{\nabla }\;\cdot \;\left(\sigma \frac{\partial \vec{A}}{\partial t}\right)=0 \end{array}$$

which is the transient representation of the MQS A-ϕ formulation. In this case, $\vec{A}$ and ϕ are coupled, and the effects of the conductively induced currents on $\vec{A}$ are considered. However, due to the complexity of numerical computation of the time-dependent functions ($\vec{A}\left(r,t\right)$ and ϕ(r,t)), it is more usual to use the harmonic expressions of Equations (28a) and (28b):

(29a) $$\begin{array}{cc} \; & j\omega \sigma \vec{A}+\sigma \vec{\nabla }\phi +\frac{1}{\mu_{0}}\vec{\nabla }\times \vec{\nabla }\times \vec{A}={\vec{J}}_{s} \end{array}$$

(29b) $$\begin{array}{cc} \; & j\omega \vec{\nabla }\;\cdot \;\left(\sigma \vec{A}\right)+\vec{\nabla }\;\cdot \;\left(\sigma \vec{\nabla }\phi \right)=0 \end{array}$$

The harmonic formulation can be used considering that the stimulation current presents a constant frequency (ω). This approximation should be used carefully, since the stimulation peak patterns are far from having a harmonic waveform. It seems unnecessary and a waste of computational resources to consider the induction currents for TMS. However, this approach could be useful for optimizing the effect of electric conductivity and geometry of the coil on the TMS. It could also be used for analyzing TMS in subjects with non-normative brains, such as those with brain implants.

### 3.5. TMS Full Maxwell Equation Formulation and the Darwin Model

The numerical computation of the electric field can be performed without any quasistatic approximation using Equations (7) and (8), but again, the complexity of the numerical computation of these equations is substantial. Therefore, the harmonic formulation of these equations is more commonly used:

(30a) $$\begin{array}{cc} \; & \left(j\omega \sigma -\omega^{2}\epsilon_{o}\epsilon_{r}\right)\vec{A}+\left(\sigma +j\omega \epsilon_{o}\epsilon_{r}\right)\vec{\nabla }\phi +\frac{1}{\mu_{0}}\vec{\nabla }\times \vec{\nabla }\times \vec{A}={\vec{J}}_{s} \end{array}$$

(30b) $$\begin{array}{cc} \; & \vec{\nabla }\;\cdot \;\left(\left(j\omega \sigma -\omega^{2}\epsilon_{o}\epsilon_{r}\right)\vec{A}\right)+\vec{\nabla }\;\cdot \;\left(\left(\sigma +j\omega \epsilon_{o}\epsilon_{r}\right)\vec{\nabla }\phi \right)=\nabla \;\cdot \;\vec{J_{s}} \end{array}$$

Nevertheless, apart from the harmonic stimulus limitation, this formulation still presents high computation complexity. An alternative that could be used for transient process computation is the Darwin model [61]. The Darwin model is not a full Maxwell equation formulation but allows for consideration of almost all the coupled effects, except for wave propagation. Since the domain scale in TMS is significantly smaller than the wavelength of the excitation magnetic field, simulation of wave propagation is completely unnecessary. The Darwin model is based on Helmholtz decomposition [45], in which the electric field is decomposed into an irrotational component and a solenoid component: $\vec{E}={\vec{E}}_{irr}+{\vec{E}}_{sol}$, where $\vec{\nabla }\times {\vec{E}}_{irr}=0$ and $\vec{\nabla }\;\cdot \;{\vec{E}}_{sol}=0$. After replacing the electric field in the Maxwell equations with these approximations, neglecting the radiation effects and replacing the expression of the potentials yields:

(31a) $$\begin{array}{cc} \; & \vec{\nabla }\times \left(\frac{1}{\mu }\vec{\nabla }\times \vec{A}\right)+\sigma \left(\frac{\partial A}{\partial t}+\vec{\nabla }\phi \right)+\frac{\partial }{\partial t}\left(\epsilon \vec{\nabla }\phi \right)={\vec{J}}_{s} \end{array}$$

(31b) $$\begin{array}{cc} \; & \vec{\nabla }\;\cdot \;\left(\sigma \left(\frac{\partial \vec{A}}{\partial t}+\vec{\nabla }\phi \right)\right)+\vec{\nabla }\;\cdot \;\frac{\partial }{\partial t}\left(\epsilon \vec{\nabla }\phi \right)=\nabla \;\cdot \;{\vec{J}}_{s} \end{array}$$

This formulation takes into account all the effects relevant for TMS included in the full Maxwell equation formulation, and it is less computationally complex than integrating the full Maxwell equations. Moreover, in this case, it seems excessive to include capacitive effects for TMS simulations, but it could be useful to optimize the stimulation devices and for analysis of TMS in subjects with non-normative brains, such as those with brain implants.

### 3.6. Boundary Value Problems of TMS

**Figure 1:**
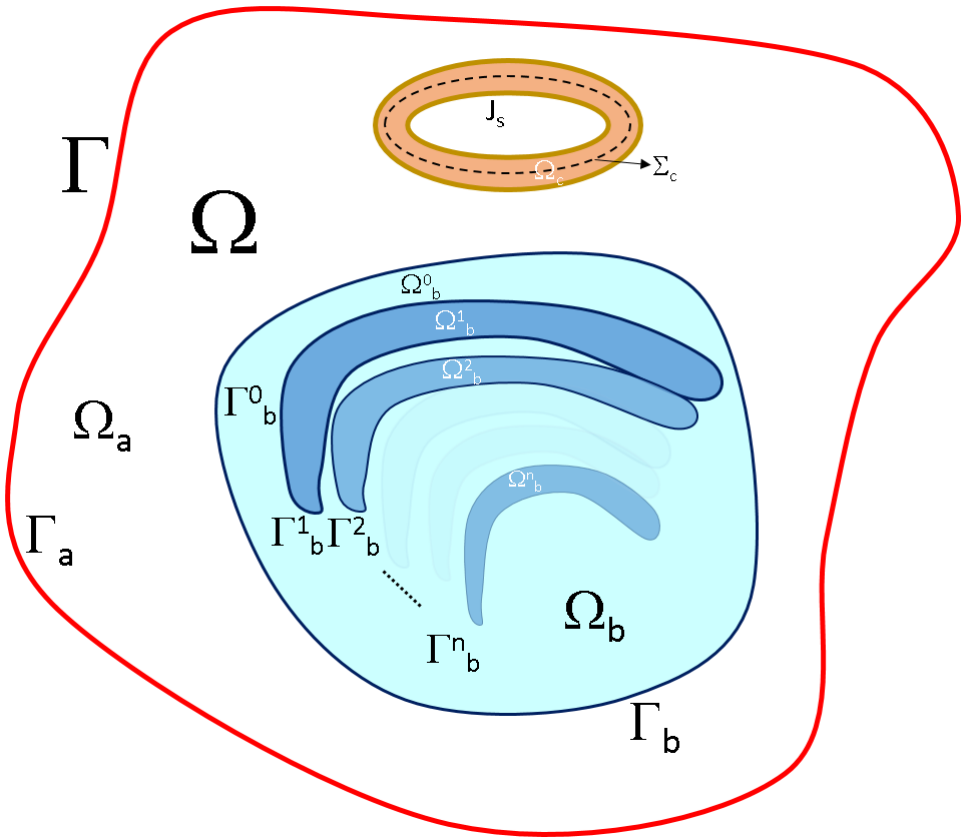
Domains and domain boundaries for TMS boundary value problems.

Figure 1 shows the domain and boundary representation of a generic TMS setup. The meaning of the domains, their boundaries, and electric and magnetic properties are shown in Table 1.

The coil is a special domain, as the source of external current, the electric conductivity of which depends on the type of quasistatic formulation. In this case, it is not the boundary that is relevant but the integration path used to compute the magnetic potential vector, as shown latter.

Based on these assumptions related to the domains, the boundary value problems(BVP) can be expressed as follows:

*The Laplace MQS-ϕ BVP:*

(32a) $$\begin{array}{cccc} \; & {\vec{\nabla }}^{2}\vec{A}=-\mu_{0}{\vec{J}}_{s} & \; & \mathrm{on}\;\Omega \end{array}$$

(32b) $$\begin{array}{cccc} \; & {\vec{\nabla }}^{2}\phi =0 & \; & \mathrm{on}\;\Omega_{b} \end{array}$$

(32c) $$\begin{array}{cccc} \; & \vec{\nabla }\times \vec{A}\;\cdot \;n=0 & \; & \mathrm{on}\;\Gamma \end{array}$$

(32d) $$\begin{array}{cccc} \; & \vec{\nabla }\phi \;\cdot \;n=-\frac{\partial \vec{A}}{\partial t}\;\cdot \;n & \; & \mathrm{on}\;\Gamma_{b} \end{array}$$

*The Poisson MQS-ϕ BVP:*

(33a) $$\begin{array}{cccc} \; & {\vec{\nabla }}^{2}\vec{A}=-\mu_{0}{\vec{J}}_{s} & \; & \mathrm{on}\;\Omega \end{array}$$

(33b) $$\begin{array}{cccc} \; & \vec{\nabla }\;\cdot \;\left(\sigma \vec{\nabla }\phi \right)=-\vec{\nabla }\;\cdot \;\left(\sigma \frac{\partial \vec{A}}{\partial t}\right)\; & \; & \mathrm{on}\;\Omega_{b} \end{array}$$

(33c) $$\begin{array}{cccc} \; & \vec{\nabla }\times \vec{A}\;\cdot \;n=0 & \; & \mathrm{on}\;\Gamma \end{array}$$

(33d) $$\begin{array}{cccc} \; & \vec{\nabla }\phi \;\cdot \;n=0 & \; & \mathrm{on}\;\Gamma \end{array}$$

*The MQS A-ϕ BVP:*

(34a) $$\begin{array}{cccc} \; & \sigma \left(\frac{\partial \vec{A}}{\partial t}+\nabla \phi \right)-\frac{1}{\mu_{0}}\nabla^{2}\vec{A}={\vec{J}}_{s} & \; & \mathrm{on}\;\Omega \end{array}$$

(34b) $$\begin{array}{cccc} \; & \vec{\nabla }\;\cdot \;\left(\sigma \vec{\nabla }\phi \right)=-\vec{\nabla }\;\cdot \;\left(\sigma \frac{\partial \vec{A}}{\partial t}\right)\; & \; & \mathrm{on}\;\Omega_{b} \end{array}$$

(34c) $$\begin{array}{cccc} \; & \vec{\nabla }\times \vec{A}\;\cdot \;n=0 & \; & \mathrm{on}\;\Gamma \end{array}$$

(34d) $$\begin{array}{cccc} \; & \vec{\nabla }\phi \;\cdot \;n=0 & \; & \mathrm{on}\;\Gamma \end{array}$$

*The Darwin model BVP:*

(35a) $$\begin{array}{cccc} \; & \vec{\nabla }\times \left(\frac{1}{\mu }\vec{\nabla }\times \vec{A}\right)+\sigma \left(\frac{\partial A}{\partial t}+\vec{\nabla }\phi \right)+\frac{\partial }{\partial t}\left(\epsilon \vec{\nabla }\phi \right)={\vec{J}}_{s}\; & \; & \mathrm{on}\;\Omega \end{array}$$

(35b) $$\begin{array}{cccc} \; & \vec{\nabla }\;\cdot \;\left(\sigma \left(\frac{\partial \vec{A}}{\partial t}+\vec{\nabla }\phi \right)\right)+\vec{\nabla }\;\cdot \;\frac{\partial }{\partial t}\left(\epsilon \vec{\nabla }\phi \right)=\nabla \;\cdot \;{\vec{J}}_{s}\; & \; & \mathrm{on}\;\Omega \end{array}$$

(35c) $$\begin{array}{cccc} \; & \vec{\nabla }\times \vec{A}\;\cdot \;n=0 & \; & \mathrm{on}\;\Gamma \end{array}$$

(35d) $$\begin{array}{cccc} \; & \vec{\nabla }\phi \;\cdot \;n=0 & \; & \mathrm{on}\;\Gamma \end{array}$$

Several works have used the MQS ϕ formulation. Table 2 lists some of these works and their models:

## 4. Solutions of TMS Field BVPs

### 4.1. Solution of MQS-ϕ BVP

Various methods have been proposed to address the MQS-ϕ formulation. In the case of the Laplace MQS-ϕ formulation, one of the most common solution is, first, to compute the primary field, $E_{p}=-\frac{\partial \vec{A}}{\partial t}$, where $\vec{A}$ is obtained by integrating the Poisson Equation (32a) over the volume of the coil ($\Omega_{coil}$), which gives:

(36) $$\vec{A}\left(r_{o},t\right)=\frac{\mu_{o}}{4\pi }\int_{\Omega_{coil}}^{}\frac{{\vec{J}}_{s}\left(r,t\right)}{\left|r-r_{o}\right|}dV$$

where ${\vec{J}}_{s}\left(r,t\right)$ is the current density. This equation is easy to solve numerically. For example, in the case of a circular coil with uniform current density ($J_{s}\left(t\right)$), a constant cross-section area (*S*) and a current path in a plane perpendicular to the z axis in the domain (Ω), each component of the magnetic potential vector at the position of $r=r_{o}$, $\vec{A}\left(r_{o},t\right)$ could be computed as as:

(37) $$\vec{A}\left(r_{o},t\right)=\frac{\mu_{o}SJ_{s}\left(t\right)}{4\pi }\oint_{\Sigma_{coil}}^{}\frac{1}{\left|r-r_{o}\right|}d\vec{l}$$

where $\Sigma_{coil}$ is the path. Equation (37) can be numerically computed as follows:

(38a) $$\begin{array}{cc} \; & A_{x}\left(r_{o},t\right)=\frac{\mu_{o}SJ_{s}\left(t\right)}{4\pi }\sum_{i=0}^{N-1}\frac{\Delta x_{i}}{\sqrt{{\left(x_{i}-x_{o}\right)}^{2}+{\left(y_{i}-y_{o}\right)}^{2}+{\left(z_{i}-z_{o}\right)}^{2}}} \end{array}$$

(38b) $$\begin{array}{cc} \; & A_{y}\left(r_{o},t\right)=\frac{\mu_{o}SJ_{s}\left(t\right)}{4\pi }\sum_{i=0}^{N-1}\frac{\Delta y_{i}}{\sqrt{{\left(x_{i}-x_{o}\right)}^{2}+{\left(y_{i}-y_{o}\right)}^{2}+{\left(z_{i}-z_{o}\right)}^{2}}} \end{array}$$

(38c) $$\begin{array}{cc} \; & A_{z}\left(r_{o},t\right)=0,\forall r_{o}\;\epsilon \;\Omega \end{array}$$

$$\forall \vec{r}=\left(x_{i},y_{i},z_{i}\right)\;\epsilon \;\Omega ,\;r\neq r_{o}$$

where

(39a) $$\begin{array}{cc} \; & x_{i}=X_{m}+R\cos\left(\psi_{i}\right) \end{array}$$

(39b) $$\begin{array}{cc} \; & y_{i}=Y_{m}+R\sin\left(\psi_{i}\right) \end{array}$$

and

(40) $$\Delta x_{i}=x_{i+1}-x_{i},\;\Delta y_{i}=y_{i+1}-y_{i}$$

where *S*, $X_{m}$ and $Y_{m}$ are the coil cross-sectional area, the coil center position on the x axis and the coil center position on the y axis, respectively. It is possible to set up different types of excitation coils by adding coils with different parameters, positions and sizes.

Once the primary field is obtained, the secondary field can be computed using the following equation: $E_{s}=-\nabla \phi$, where ϕ is obtained after solving the Laplace Equation (32b) and the boundary condition (32d).

In the case of the Poisson MQS-ϕ formulation, $\vec{A}$ is computed using the same procedure as in the Laplace formulation, but ϕ is computed by solving the Poisson Equation (33b) with boundary conditions (33c) and (33d). This equation can be solved using numerical, analytical or semianalytical methods depending on the complexity of the brain model. Two of the most commonly used numerical methods are the finite difference method (FDM) [63,64,65,66,67] and the finite element method (FEM) [64,68,69,70].

Equation (33b) for the scalar potential can be expressed as:

(41) $$\vec{\nabla }\;\cdot \;\left(\sigma \nabla \phi \right)=\vec{\nabla }\sigma \;\cdot \;\vec{\nabla }\phi +\sigma \;\cdot \;\nabla^{2}\phi =\vec{\nabla }\;\cdot \;{\vec{J}}_{ind}$$

where ${\vec{J}}_{ind}=\sigma \vec{E}=-\sigma \frac{\partial \vec{A}}{\partial t}$ is the conductively induced current.The FDM to solve this Poisson equation can be implemented using the interactive method:

(42) $$\begin{array}{c} \phi_{i,j,k}=\frac{1}{\alpha }[\frac{\sigma_{i-\frac{1}{2},j,k}\phi_{i-1,j,k}+\sigma_{i+\frac{1}{2},j,k}\phi_{i+1,j,k}}{\Delta x^{2}}+\frac{\sigma_{i,j-\frac{1}{2},k}\phi_{i,j-1,k}+\sigma_{i,j+\frac{1}{2},k}\phi_{i,j+1,k}}{\Delta y^{2}}+\\ \frac{\sigma_{i,j,k-\frac{1}{2}}\phi_{i,j,k-1}+\sigma_{i,j,k+\frac{1}{2}}\phi_{i,j,k+1}]}{\Delta z^{2}}+\\ \frac{1}{2\alpha }\left[\frac{\left(J_{i+1,j,k}^{x}-J_{i-1,j,k}^{x}\right)}{\Delta x}+\frac{\left(J_{i,j+1,k}^{y}-J_{i,j-1,k}^{y}\right)}{\Delta y}+\frac{\left(J_{i,j,k+1}^{z}-J_{i,j,k-1}^{z}\right)}{\Delta z}\right] \end{array}$$

where $\alpha =\left[\frac{\sigma_{i-\frac{1}{2},j,k}+\sigma_{i+\frac{1}{2},j,k}}{\Delta x^{2}}+\frac{\sigma_{i,j-\frac{1}{2},k}+\sigma_{i,j+\frac{1}{2},k}}{\Delta y^{2}}+\frac{\sigma_{i,j,k-\frac{1}{2}}+\sigma_{i,j,k+\frac{1}{2}}}{\Delta z^{2}}\right]$ and $J_{i,j,k}^{x}=\sigma_{i,j,k}E_{i,j,k}^{x}$, $J_{i,j,k}^{y}=\sigma_{i,j,k}E_{i,j,k}^{y}$, $J_{i,j,k}^{z}=\sigma_{i,j,k}E_{i,j,k}^{z}$ are the currents densities due to the primary electric field ($E_{p}=\left(E_{p}^{x},E_{p}^{y},E_{p}^{z}\right)$), and Δx, Δy and Δz are the grid intervals. This method is valid for σ≠0.

It is clear that in the case of the Laplace MQS-V formulation, the same approach as before could be used, except that ${\vec{J}}_{ind}=0$,

(43) $$\phi_{i,j,k}=\frac{1}{6}\left(\phi_{i-1,j,k}+\phi_{i+1,j,k}+\phi_{i,j-1,k}+\phi_{i,j+1,k}+\phi_{i,j,k-1}+\phi_{i,j,k+1}\right)$$

This expression should be solved using the boundary conditions (32d), which can be expressed as:

(44) $$\frac{\partial \phi }{\partial x}n_{x}+\frac{\partial \phi }{\partial y}n_{y}+\frac{\partial \phi }{\partial z}n_{z}=E_{x}^{p}n_{x}+E_{y}^{p}n_{y}+E_{x}^{p}n_{z}$$

which is solved using the finite difference as: $\phi_{i,j,k}=\frac{1}{\frac{n_{x}}{\Delta x}+\frac{n_{y}}{\Delta y}+\frac{n_{z}}{\Delta z}}\left(E_{x}^{p}+\frac{\phi_{i-1,j,k}}{\Delta x}\right)n_{x}+$$\left(E_{y}^{p}+\frac{\phi_{i,j-1,k}}{\Delta y}\right)n_{y}+\left(E_{z}^{p}+\frac{\phi_{i,j,k-1}}{\Delta z}\right)n_{z}$

It is also important to note that the above numerical solution corresponds to an Euclidean coordinate system, and consequently, the brain should be circumscribed inside an air cube, and the boundary conditions in this case correspond to an interior boundary. From a computational coding perspective, the FDM is straightforward. However, the precision and the computation time required by this method depend on the complexity of the geometry.

#### 4.1.1. Solutions Using FEM

The FEM usually requires much less computational time than the FDM but can be more tricky to implement. The Poisson MQS-ϕ BVP can be solved with FEM using different approaches: the direct minimization of energy functional and the weighted residuals or the Galerkin method. For example, Weiping Wang and Solomon R. Eisenber [95] considered the following energy functional:

(45) $$W\left(\phi \right)=\int \left(\vec{J}\;\cdot \;\vec{E}\right)dV,$$

which represents the energy dissipated in the conduction media during the induction process. Using the expressions of the induced current and electric fields, the resultant equation is:

(46) $$W\left(\phi \right)=\int \sigma \left(\vec{\nabla }\phi +\frac{\partial \vec{A}}{\partial t}\right)\;\cdot \;\left(\vec{\nabla }\phi +\frac{\partial \vec{A}}{\partial t}\right)dV$$

The objective is to find the potentials (ϕ and $\vec{A}$) that minimize the energy functional, i.e., W(ϕ). The potentials at any coordinate (x,y,z) inside the domain can be obtained from the potential at the nodes of the finite elements (usually tetrahedral elements), in which the domain is divided using interpolation functions:

(47a) $$\begin{array}{cc} \; & \Phi^{e}\left(x,y,z\right)=\sum_{i=1}^{n_{n}}\phi_{i}^{e}N_{i}\left(x,y,z\right) \end{array}$$

(47b) $$\begin{array}{cc} \; & \vec{A^{e}}\left(x,y,z\right)=\sum_{i=1}^{n_{n}}{\vec{A^{e}}}_{i}N_{i}\left(x,y,z\right) \end{array}$$

where $n_{n}$ is the number of nodes of the finite elements, $n_{n}=4$ represents tetrahedral elements, $N_{i}\left(x,y,z\right)$ are the interpolation functions and $\phi_{i}^{e}$, ${\vec{A^{e}}}_{i}={\left(A^{x}\right)}_{i}^{e}\vec{i}+{\left(A^{y}\right)}_{i}^{e}\vec{j}+{\left(A^{z}\right)}_{i}^{e}\vec{k}$ are the electric scalar potential and the spacial part of the magnetic vector potential, respectively, corresponding to node *i* in element *e*. In this case, variable separation for the magnetic potential vector ($\vec{A}\left(x,y,z,t\right)=\vec{A}\left(x,y,z\right)\;\cdot \;f\left(t\right)$) is assumed. The interpolation functions are usually linear functions of the form $N_{i}\left(x_{1},x_{2},\ldots ,x_{n_{n}}\right)=\sum_{j=1}^{n_{n}}a_{i}^{j}x_{j}+d$. For example, in the case of tetrahedral elements ($N_{i}\left(x,y,z\right)=a_{i}x+b_{i}y+c_{i}z+d$), the coefficients are obtained by solving a linear equations system, considering that $N_{i}\left(x,y,z\right)=1$ for $x=x_{i},y=y_{i},z=z_{i}$ and $N_{i}\left(x,y,z\right)=0$ for $x=x_{j},y=y_{j},z=z_{j}$, where $x_{i},y_{i},z_{i}$ are the coordinates of node *i*, and $x_{j},y_{j},z_{j}$ are the coordinates of the other nodes of the element.

The integral corresponding to total dissipated power for each element given by Equation (46) is divided in two parts: a voltage-dependent power and a constant term:

(48) $$W^{e}\left(\Phi^{e}\right)=W_{N}^{e}\left(\Phi^{e}\right)+W_{const}^{e}$$

Considering that $\frac{\partial \vec{A}}{\partial t}=\dot{f}\vec{A}\left(x,y,z\right)$, $\dot{f}=\frac{\partial f(t)}{\partial t}$.

(49) $$W_{N}^{e}\left(\Phi^{e}\right)=\int \left(\sigma {\left(\nabla \Phi^{e}\right)}^{2}+2\sigma \dot{f}\vec{A}\;\cdot \;\vec{\nabla }\Phi^{e}\right)dV$$

(50) $$W_{const}^{e}\left(\Phi^{e}\right)={\left(\dot{f}\right)}^{2}\int \sigma {\left(\vec{A}\right)}^{2}dV$$

The constant term does not influence the minimum value of the functional and is therefore removed. The potential dependent term after replacing Equations (47a) and (47b) gives:

(51) $$W_{N}^{e}\left(\Phi^{e}\right)={\left(\Phi^{e}\right)}^{T}P^{e}\Phi^{e}+{\left(\Phi^{e}\right)}^{T}Q^{e}$$

where $\Phi^{e}={\left(\phi_{1}^{e},\phi_{2}^{e},\phi_{3}^{e},\phi_{4}^{e},\ldots ,\phi_{n_{n}}^{e}\right)}^{T}$ is the vector of scalar potentials corresponding to the nodes of the element (*e*), $P^{e}$ is a square matrix and $Q^{e}$ is a column vector given by:

(52) $$P_{ij}^{e}=\int \sigma \nabla N_{i}\;\cdot \;\nabla N_{j}dV$$

and

(53) $$Q_{i}^{e}=2\dot{f}\sum_{j=1}^{n_{n}}\int \sigma \nabla N_{i}\;\cdot \;{\vec{A}}_{j}N_{j}dV$$

These integrals are very easy to compute, since the interpolation functions are linear functions. For example, in the case of tetrahedral elements, the integrated expressions are:

(54) $$P_{ij}^{e}=V^{e}\left(\Lambda_{i}^{x}a_{j}+\Lambda_{i}^{y}b_{j}+\Lambda_{i}^{z}c_{j}\right)$$

and

(55) $$Q_{i}^{e}=\frac{2\dot{f}V^{e}}{2}\sum_{j=1}^{4}\left(\Lambda_{i}^{x}A_{j}^{x}+\Lambda_{i}^{y}A_{j}^{y}+\Lambda_{i}^{z}A_{j}^{z}\right)$$

where $V^{e}$ is the volume of the element, and $\Lambda_{i}^{k}=\sigma_{kx}a_{i}+\sigma_{ky}b_{i}+\sigma_{kz}c_{i}$, k=x,y,z. Equation (51) corresponds to the power dissipated in one element. The total power is obtained as the sum of the power of all elements:

(56) $$W_{N}\left(\Phi \right)=\sum_{e}W_{N}^{e}\left(\Phi^{e}\right)$$

Nevertheless, it is worth noting that to obtain this sum, the dimensions of the vectors ($\Phi^{e}$ and $Q^{e}$) and the matrix ($P^{e}$) should be increased such that $dim\left(\Phi^{e}\right)=n_{n}\times N_{e}$, $dim\left(Q^{e}\right)=n_{n}\times N_{e}$ and $dim\left(P^{e}\right)={\left(n_{n}\times N_{e}\right)}^{2}$, filling the rest of the components with zero and shifting the position of the vector or matrix according to the element index (*e*) and the number of nodes $n_{n}$. For example, the new vector ($\Phi^{e}$) should look like:

(57) $$\Phi^{e}={\left(\overset{\left(e-1\right)\times n_{n}}{\overset{︷}{0,\ldots ,0}},\phi_{1}^{e},\phi_{2}^{e},\phi_{3}^{e},\phi_{4}^{e},\ldots ,\phi_{n_{n}}^{e},0,\ldots ,0\right)}^{T}$$

Similarly to the vector ($Q^{e}$), the matrix ($P^{e}$) should look like:

(58) $$\begin{array}{c} P^{e}=\left[\begin{array}{ccccc} \overset{\left(e-1\right)\times n_{n}+1}{\overset{︷}{\begin{array}{ccc} 0 & \ldots & 0 \end{array}}} & \cdots & 0 & \; & 0\\ \vdots \;\vdots & \; & \vdots & \; & \vdots \\ \; & \; & \; & \; & \\ \;0\;\cdots \;P_{11}^{e} & \cdots & P_{14}^{e} & \cdots & 0\\ \vdots \;\vdots & \; & \vdots & \; & \vdots \\ \;0\;\cdots \;P_{41}^{e} & \cdots & P_{44}^{e} & \cdots & 0\\ \vdots \;\vdots & \; & \vdots & \; & \vdots \\ \;0\; & \cdots & \; & & 0 \end{array}\right]\begin{array}{cc} \left.\right\}\left(e-1\right)\times n_{n}+1\\ \; & \;\\ \; & \;\\ \; & \;\\ \; & \; \end{array} \end{array}$$

In the new space, which is called disjoint space, Equation (56) can be written as:

(59) $$W_{dis}\left(\Phi_{dis}\right)=\Phi_{dis}^{T}P_{dis}\Phi_{dis}+\Phi_{dis}^{T}Q_{dis}$$

The potentials in the disjointed space are represented by variables refereed to each element, which gives a large number of variables. The number of variables can be reduced significantly by considering that neighbor elements share nodes, and therefore, it is possible to use the same variable for the potential of the nodes shared by different elements. In other words, the variables can be renamed using a common global notation for all the nodes inside the domain. This process is called assembly and can be performed using a connectivity matrix (C) such that:

(60) $$\Phi_{dis}=C\Phi_{asem}$$

Substituting this equation into Equation (69) gives:

(61) $$W_{asem}\left(\Phi_{asem}\right)=\Phi_{asem}^{T}P\Phi_{asem}+\Phi_{asem}^{T}Q$$

where $P=CP_{dis}C^{T}$ and $Q=CQ_{dis}$.

The linear equation system used to obtain the potentials at each node ($\Phi_{i}$) is formed by finding the minimum of the energy with respect to each node potential:

(62) $$\frac{\partial W(\Phi )}{\partial \Phi_{i}}=0$$

which results in the following linear equation system:

(63) 2PΦ+Q=0

Furthermore, to obtain a unique solution, it is necessary to impose an additional condition, which is usually achieved nu setting the potential of the Nth node to zero ($\Phi_{N}=0$). Thus, the equation system is updated by removing the Nth row and column of matrix P and the last element of *Q*, resulting in a matrix ($P^{\prime }$ ($dim\left(P^{\prime }\right)=\left(N-1\right)\times \left(N-1\right)$)) and a vector ($Q^{\prime }$ ($dim\left(Q^{\prime }\right)=1\times \left(N-1\right)$)). The solution for the equations system is:

(64) $$\Phi =-\frac{1}{2}P^{\prime }-1Q^{\prime }$$

After computing the scalar potential, it is possible to obtain the electric field using Equation (6).

#### 4.1.2. Weighted Residual Galerkin Method

The Weighted residual Galerkin method [68,69] is one of the most widely used FEMs. In this case, the starting point is Equation (32), which considers the domain of a single element ($\Omega_{e}$), and the weak formulation [68,69] and the residual for the element (*e*) can be written as:

(65) $$r_{e}=\vec{\nabla }\;\cdot \;\left(\sigma \vec{\nabla }\phi \right)+\vec{\nabla }\;\cdot \;\left(\sigma \frac{\partial \vec{A}}{\partial t}\right)$$

If the numerical solution were exact, the residual would be zero. However, since the numerical solutions are not ideal, it is necessary to find a solution that minimizes the residual or, in the case of this method, the weighted residual. The procedure involves multiplying the residual by a weight (*w*), integrating this product over the volume of the element and setting the integral to zero.

(66) $$\int_{\Omega_{e}}w\;r_{e}\;dV=\int_{\Omega_{e}}w\left(\vec{\nabla }\;\cdot \;\left(\sigma \vec{\nabla }\phi \right)+\vec{\nabla }\;\cdot \;\left(\sigma \frac{\partial \vec{A}}{\partial t}\right)\right)dV=0$$

using the identity $\vec{\nabla }\;\cdot \;\left(w\vec{A}\right)=w\vec{\nabla }\;\cdot \;\vec{A}+\vec{A}\;\cdot \;\left(\vec{\nabla }w\right)$, which implies that:

(67) $$w\vec{\nabla }\;\cdot \;\vec{A}=\vec{\nabla }\;\cdot \;\left(w\vec{A}\right)-\vec{A}\;\cdot \;\left(\vec{\nabla }w\right)$$

Using this identity, Equation (67) can be modified as:

(68) $$\int_{\Omega_{e}}\vec{\nabla }\;\cdot \;\left(w\sigma \vec{\nabla }\phi \right)dV-\int_{\Omega_{e}}\left(\sigma \vec{\nabla }\phi \right)\;\cdot \;\vec{\nabla }w\;dV+\int_{\Omega_{e}}w\vec{\nabla }\;\cdot \;\left(\sigma \frac{\partial \vec{A}}{\partial t}\right)dV=0$$

Applying the divergence theorem [45] ($\int_{\Omega_{e}}\left(\nabla \;\cdot \;\vec{F}\right)dV=\oint_{\Gamma_{e}}\left(\vec{F}\;\cdot \;n\right)dS$) to the first term of Equation (68) and rearranging the equation yields:

(69) $$-\int_{\Omega_{e}}\left(\sigma \vec{\nabla }\phi \right)\;\cdot \;\vec{\nabla }w\;dV+\int_{\Omega_{e}}w\vec{\nabla }\;\cdot \;\left(\sigma \frac{\partial \vec{A}}{\partial t}\right)dV+\oint_{\Gamma_{e}}w\left(\sigma \vec{\nabla }\phi \right)\;\cdot \;ndS=0$$

The three components of this equation are the potential-dependent term, the source term and the boundary condition term, in that order. The source term can be modified by considering the induced current density ($\vec{J}=-\vec{\sigma }\;\cdot \;\frac{\partial \vec{A}}{\partial t}$):

(70) $$-\int_{\Omega_{e}}\left(\sigma \vec{\nabla }\phi \right)\;\cdot \;\vec{\nabla }w\;dV-\int_{\Omega_{e}}w\vec{\nabla }\;\cdot \;\vec{J}dV+\oint_{\Gamma_{e}}w\left(\sigma \vec{\nabla }\phi \right)\;\cdot \;ndS=0$$

According to the Garlerkin formulation [68], the weight function (*w*) should be set equal to the interpolation functions:

(71) $$w=N_{i}^{e}\left(x,y,z\right)=a_{i}x+b_{i}y+c_{i}z+d$$

Moreover, using Equation (47a) for the interpolation of the scalar potential ($\phi_{e}$) and considering the interpolation for the induced current density values:

(72) $$\vec{J^{e}}\left(x,y,z\right)=\sum_{i=1}^{n_{n}}J_{i}^{e}N_{i}\left(x,y,z\right)$$

Equation (70) becomes a system of $n_{n}$ equations, where $n_{n}$ is the number of element nodes:

(73) $$P^{e}\Phi^{e}+Q^{e}J^{e}+B^{e}=0$$

where

(74a) $$\begin{array}{cc} \; & P_{ij}^{e}=\int_{\Omega_{e}}\left[\sigma_{xx}^{e}\frac{\partial N_{i}}{\partial x}\frac{\partial N_{j}}{\partial x}+\sigma_{yy}^{e}\frac{\partial N_{i}}{\partial y}\frac{\partial N_{j}}{\partial y}+\sigma_{zz}^{e}\frac{\partial N_{i}}{\partial z}\frac{\partial N_{j}}{dz}\right]dV \end{array}$$

(74b) $$\begin{array}{cc} \; & Q_{ij}^{e}=\int_{\Omega_{e}}N_{i}\left[\frac{\partial N_{j}}{\partial x}+\frac{\partial N_{j}}{\partial y}+\frac{\partial N_{j}}{\partial z}\right]dV \end{array}$$

(74c) $$\begin{array}{cc} \; & B_{i}^{e}=-\oint_{\Gamma_{e}}N_{i}\left[\sigma_{xx}^{e}\frac{\partial \Phi }{\partial x}n_{x}+\sigma_{yy}^{e}\frac{\partial \Phi }{\partial y}n_{y}+\sigma_{zz}^{e}\frac{\partial \Phi }{\partial z}n_{z}\right]dV \end{array}$$

$$dim\left(P^{e}\right)=n_{n}\times n_{n},dim\left(Q^{e}\right)=n_{n}\times n_{n},dim\left(J^{e}\right)=1\times n_{n},dim\left(B^{e}\right)=1\times n_{n},$$

Due to the fact that the normal vector of the faces shared by two neighbor elements has opposite directions [68,69], the integral $B^{e}$ cancels for interior element boundaries, and $B^{e}$ is different from zero only for the outer boundaries. Furthermore, if the simulation is performed considering an air box circumscribing the brain, in the air, σ=0; therefore, $B^{e}\left(x,y,z\right)=0,\forall \left(x,y,z\right)\;\epsilon \;\Omega_{sys}$. Accordingly, this term can be removed from the equation, and the equation system becomes:

(75) $$P^{e}\Phi^{e}+Q^{e}J^{e}=0$$

On the other hand, if the brain is considered the outer boundary and the approximation of having only one tissue interface, for example, the interface between the brain and the air, the second term can also be modified using the divergence theorem, which gives:

(76) $$-\int_{\Omega_{e}}\left(\sigma \vec{\nabla }\phi \right)\;\cdot \;\vec{\nabla }w\;dV+\oint_{\Gamma_{e}}w\left(\sigma \frac{\partial \vec{A}}{\partial t}\right)ndS+\oint_{\Gamma_{e}}w\left(\sigma \vec{\nabla }\phi \right)\;\cdot \;ndS=0$$

or

(77) $$-\int_{\Omega_{e}}\left(\sigma \vec{\nabla }\phi \right)\;\cdot \;\vec{\nabla }w\;dV+\oint_{\Gamma_{e}}w\left(\sigma \frac{\partial \vec{A}}{\partial t}+\sigma \vec{\nabla }\phi \right)\;\cdot \;ndS=0$$

Considering the boundary condition corresponding to Equation (32d), it is evident that the second term is zero. Therefore, for this particular case, Equation (77) becomes:

(78) $$\int_{\Omega_{e}}\left(\sigma \vec{\nabla }\phi \right)\;\cdot \;\vec{\nabla }w\;dV=0$$

which gives the following linear equation system:

(79) PΦ=0

The trivial solution (Φ=0) is avoided, considering the boundary condition expressed by Equation (32d) for boundary nodes. This can be implemented in several forms, for example, by considering the following equation system that results from condition Equation (32d):

(80a) $$\begin{array}{cc} \; & \frac{\partial \Phi }{\partial x}=-\frac{\partial A_{x}}{\partial t}=E_{x}^{p} \end{array}$$

(80b) $$\begin{array}{cc} \; & \frac{\partial \Phi }{\partial y}=E_{y}^{p} \end{array}$$

(80c) $$\begin{array}{cc} \; & \frac{\partial \Phi }{\partial z}=E_{z}^{p} \end{array}$$

The value of Φ for the boundary nodes can be obtained from these equations for each element separately, forming a 3×3 linear equation system:

(81a) $$\begin{array}{cc} \; & \sum_{i=1}^{3}\Phi_{i}^{e}\frac{\partial N_{i}^{s}}{\partial x}={\left(E_{x}^{p}\right)}^{e} \end{array}$$

(81b) $$\begin{array}{cc} \; & \sum_{i=1}^{3}\Phi_{i}^{e}\frac{\partial N_{i}^{s}}{\partial y}={\left(E_{y}^{p}\right)}^{e} \end{array}$$

(81c) $$\begin{array}{cc} \; & \sum_{i=1}^{3}\Phi_{i}^{e}\frac{\partial N_{i}^{s}}{\partial z}={\left(E_{z}^{p}\right)}^{e} \end{array}$$

where $N_{i}^{s}$ represents the interpolation functions for the potential of the nodes forming the triangular face, that is, facing the outer boundary, and ${\left(E_{x}^{p}\right)}^{e},{\left(E_{y}^{p}\right)}^{e},{\left(E_{z}^{p}\right)}^{e}$ are the components of the primary electric field in the boundary space.

Equation (75) corresponds to one element. To solve the global equation system, it is necessary to assembly the matrices for the entire domain (Ω). This can be done again by applying a procedure similar to that explained in the previous section. The dimensions of the matrices ($P^{e}$ and $Q^{e}$) and the vectors ($J^{e}$ and $\Phi^{e}$) are extended as shown in Equations (57) and (58), generating a system of linear equations in the disjointed space such that:

(82) $$P_{\mathrm{dis}}=\sum_{e\;\epsilon \;\Omega }P^{e}$$

(83) $$Q_{\mathrm{dis}}=\sum_{e\;\epsilon \;\Omega }Q^{e}$$

and

(84) $$J_{\mathrm{dis}}=\sum_{e\;\epsilon \;\Omega }J^{e}.$$

Therefore,

(85) $$P_{\mathrm{dis}}\Phi_{dis}+Q_{\mathrm{dis}}J_{dis}=0$$

There is also a redundancy of variables of the disjointed space due to the presence of shared nodes between neighbor elements. To remove this redundancy, it is necessary to assemble the matrices using an assembly process as in the previous section, which can be achieved using the connectivity matrix:

(86) $$\Phi_{dis}=C\Phi_{asem}$$

(87) $$J_{dis}=CJ_{asem}$$

and substituting in (85):

(88) $$P_{\mathrm{asem}}\Phi_{asem}+K_{asem}=0$$

where $P_{asem}=P_{dis}C$ and $K_{asem}=Q_{dis}CJ_{asem}$, and the potential can be obtained from:

(89) $$\Phi_{asem}=P_{\mathrm{asem}}^{-1}K_{asem}$$

### 4.2. Solution of MQS Φ-V BVP

#### 4.2.1. Solution Using FDM

The FDM can be applied to solve the BVP given by Equation (34) using several approaches. One of the most straightforward methods is the explicit interactive method [63], which can also be applied using different considerations. For example, in the case of isotropic conductivity, Equation (34a) can be divided in three equations corresponding to each of three coordinates in the Euclidean space. If Cartesian coordinates are used:

(90a) $$\begin{array}{cc} \; & \sigma \left(\frac{\partial A_{x}}{\partial t}+\frac{\partial \phi_{x}}{\partial x}\right)-\frac{1}{\mu_{0}}\nabla^{2}A_{x}=\vec{J_{x}^{s}} \end{array}$$

(90b) $$\begin{array}{cc} \; & \sigma \left(\frac{\partial A_{y}}{\partial t}+\frac{\partial \phi_{y}}{\partial y}\right)-\frac{1}{\mu_{0}}\nabla^{2}A_{y}=\vec{J_{y}^{s}} \end{array}$$

(90c) $$\begin{array}{cc} \; & \sigma \left(\frac{\partial A_{z}}{\partial t}+\frac{\partial \phi_{z}}{\partial z}\right)-\frac{1}{\mu_{0}}\nabla^{2}A_{z}=\vec{J_{z}^{s}} \end{array}$$

The procedure to obtain the electric field is described as follows:

1. For t=0, $\vec{J}=0$; therefore, $\Phi ,\vec{A}=0$;
2. For $t=t_{1}$, $\vec{A}$ is obtained from Equation (90) using the explicit interactive method in an isotropic grid (Δx=Δy=Δz=Δh) as follows:
  (91) $$\begin{array}{c} \sigma_{i,j,k}\left(\frac{{\left(A_{x}^{t}\right)}_{i,j,k}-{\left(A_{x}^{t-\Delta t}\right)}_{i,j,k}}{\Delta t}\right)-\frac{1}{\mu_{0}}\left(\frac{{\left(A_{x}^{t}\right)}_{i-1,j,k}+{\left(A_{x}^{t}\right)}_{i+1,j,k}+{\left(A_{x}^{t}\right)}_{i,j-1,k}}{{\left(\Delta h\right)}^{2}}\right)\\ +\frac{6}{\mu_{0}}\frac{{\left(A_{x}^{t}\right)}_{i,j,k}}{{\left(\Delta h\right)}^{2}}-\frac{1}{\mu_{0}}\left(\frac{{\left(A_{x}^{t}\right)}_{i,j+1,k}+{\left(A_{x}^{t}\right)}_{i,j,k-1}+{\left(A_{x}^{t}\right)}_{i,j,k+1}}{{\left(\Delta h\right)}^{2}}\right)\\ +\sigma_{i,j,k}\left(\frac{{\left(\Phi_{x}^{t-\Delta t}\right)}_{i+1,j,k}-{\left(\Phi_{x}^{t-\Delta t}\right)}_{i-1,j,k}}{2\Delta h}\right)={\left(J_{x}^{s}\right)}_{i,j,k} \end{array}$$
  which, resolved with respect to ${\left(A_{x}^{t}\right)}_{i,j,k}$, gives:
  (92) $$\begin{array}{c} {\left(A_{x}^{t}\right)}_{i,j,k}=\frac{1}{\alpha }[\beta \left({\left(A_{x}^{t}\right)}_{i-1,j,k}+{\left(A_{x}^{t}\right)}_{i+1,j,k}+{\left(A_{x}^{t}\right)}_{i,j-1,k}\right)+\\ {\left(A_{x}^{t}\right)}_{i,j+1,k}+{\left(A_{x}^{t}\right)}_{i,j,k-1}+{\left(A_{x}^{t}\right)}_{i,j,k+1}]+\\ \frac{\gamma }{\alpha }\left({\left(\Phi_{x}^{t-\Delta t}\right)}_{i+1,j,k}-{\left(\Phi_{x}^{t-\Delta t}\right)}_{i-1,j,k}\right)+\frac{\theta }{\alpha }{\left(A_{x}^{t-\Delta t}\right)}_{i,j,k}+\frac{1}{\alpha }{\left(J_{x}^{s}\right)}_{i,j,k} \end{array}$$
  where $\alpha =\left(\frac{\sigma_{i,j,k}}{\Delta t}+\frac{6}{\mu_{o}{\left(\Delta h\right)}^{2}}\right)$, $\beta =\frac{1}{\mu_{o}{\left(\Delta h\right)}^{2}}$, $\gamma =-\left(\frac{\sigma_{i,j,k}}{2\Delta h}\right)$ and $\theta =\frac{\sigma_{i,j,k}}{\Delta t}$. This equation is applied recursively to all nodes, except for the boundary nodes (where the solution is already given) and given the initial condition ($\vec{A}=0$) until the solution converges. The obtained value of $\vec{A}$ is replaced in Equation (41), which can be resolved for Φ using Equation (42);
3. The same procedure is applied to compute $A_{y}$ and $A_{z}$;
4. The values of $\vec{A}$ and Φ are used to compute the electric field using Equation (6). For the next time instant, the value of Φ is replaced in step 2, and the process is repeated.

#### 4.2.2. Solution Using FEM (Galerkin Method)

The FEM solution can also be obtained using different approaches. Considering that electric conductivity is isotropic, Equation (90) can be represented as a weighted residual as follows:

(93) $$\int_{\Omega_{e}}w\left[\sigma \left(\frac{\partial A_{x}}{\partial t}+\frac{\partial \phi }{\partial x}\right)-\frac{1}{\mu_{0}}\nabla^{2}A_{x}-\vec{J_{x}^{s}}\right]dV=0$$

or

(94) $$\int_{\Omega_{e}}\left[w\sigma \frac{\partial A_{x}}{\partial t}+w\sigma \frac{\partial \phi }{\partial x}-\frac{1}{\mu_{0}}w\nabla^{2}A_{x}-w\vec{J_{x}^{s}}\right]dV=0$$

which, applying the identity $a\nabla^{2}b=\nabla \;\cdot \;\left(a\nabla b\right)-\nabla a\;\cdot \;\nabla b$ to the third term, becomes:

(95) $$\int_{\Omega_{e}}\left[w\sigma \frac{\partial A_{x}}{\partial t}+w\sigma \frac{\partial \phi }{\partial x}-\frac{1}{\mu_{0}}\vec{\nabla }\;\cdot \;\left(w\vec{\nabla }A_{x}\right)+\frac{1}{\mu_{0}}\vec{\nabla }A_{x}\;\cdot \;\vec{\nabla }w-w\vec{J_{x}^{s}}\right]dV=0$$

Using the divergence theorem as in the previous sections applied to the third term and rearranging the equation yields:

(96) $$\begin{array}{c} \frac{1}{\mu_{0}}\int_{\Omega_{e}}\vec{\nabla }A_{x}\;\cdot \;\vec{\nabla }wdV+\int_{\Omega_{e}}\left[w\sigma \frac{\partial A_{x}}{\partial t}+w\sigma \frac{\partial \phi }{\partial x}\right]dV\\ -\int_{\Omega_{e}}w\vec{J_{x}^{s}}dV-\frac{1}{\mu_{0}}\oint_{\Gamma_{e}}w\left(\vec{\nabla }A_{x}\;\cdot \;n\right)dS=0 \end{array}$$

The potential at any position ($\left(x,y,z\right)\;\epsilon \;\Omega_{sys}$) is computed using the potential at the nodes and the interpolation functions:

(97a) $$\begin{array}{cc} \; & A_{x}^{e}\left(x,y,z\right)=\sum_{i=1}^{n_{n}}{\left(A_{x}^{e}\right)}_{i}N_{i}\left(x,y,z\right) \end{array}$$

(97b) $$\begin{array}{cc} \; & \Phi^{e}\left(x,y,z\right)=\sum_{i=1}^{n_{n}}{\left(\Phi^{e}\right)}_{i}N_{i}\left(x,y,z\right) \end{array}$$

Replacing the weight function, (*w*) with the interpolation functions in Equation (96) and $A_{x}$, ϕ with expression (97) gives:

(98) $$\begin{array}{c} P^{e}{\left(A_{x}^{t}\right)}^{e}+T^{e}{\left(A_{x}^{t}\right)}^{e}+R^{e}\Phi +T^{e}{\left(A_{x}^{t-\Delta t}\right)}^{e}+F^{e}+B^{e}=0 \end{array}$$

where $P_{ij}^{e}=\frac{1}{\mu_{o}}\int_{\Omega_{e}}\left[\frac{\partial N_{i}}{\partial x}\frac{\partial N_{j}}{\partial x}+\frac{\partial N_{i}}{\partial y}\frac{\partial N_{j}}{\partial y}+\frac{\partial N_{i}}{\partial z}\frac{\partial N_{j}}{dz}\right]dV$, $T_{ij}^{e}=-\frac{\sigma^{e}}{\Delta t}\int_{\Omega_{e}}N_{i}N_{j}dV$, $R_{ij}^{e}=$$\sigma^{e}\int_{\Omega_{e}}\frac{\partial N_{i}}{\partial x}N_{j}dV$, $F_{x}^{e}=-{\left(J_{x}^{s}\right)}^{e}\int_{\Omega_{e}}N_{i}dV$ and $B_{x}^{e}=\frac{1}{\mu_{o}}\int_{\Omega_{e}}Ni\left(\frac{\partial Ax}{\partial x}n_{x}+\frac{\partial Ax}{\partial y}n_{y}+\frac{\partial Ax}{\partial z}n_{z}\right)dV$ is the boundary condition as in the previous section.

Equation (98) corresponds to one element and can be expanded for the disjoint space using the same procedure explained in the previous two sections, after which the equation system is assembled using the connectivity matrix. The resulting equation system considering all components of $\vec{A}$ and the potential (Φ) is:

(99a) $$\begin{array}{cc} \; & \left[P+T\right]A_{x}^{t}+R\Phi =-TA_{x}^{t-\Delta t}-S_{x} \end{array}$$

(99b) $$\begin{array}{cc} \; & \left[P+T\right]A_{y}^{t}+R\Phi =-TA_{y}^{t-\Delta t}-S_{y} \end{array}$$

(99c) $$\begin{array}{cc} \; & \left[P+T\right]A_{z}^{t}+R\Phi =-TA_{z}^{t-\Delta t}-S_{z} \end{array}$$

(99d) $$\begin{array}{cc} \; & P^{\prime }\Phi =-QJ \end{array}$$

or

(100) $$\left(\begin{array}{cc} \left[P+T\right] & R\\ 0 & P^{\prime } \end{array}\right)\left(\begin{array}{c} A^{t}\\ \Phi \end{array}\right)=\left(\begin{array}{c} -TA^{t-\Delta t}-S\\ -QJ \end{array}\right)$$

where $S_{v}=F_{v}+B_{v}$ for v={x,y,z}, *J* is a row of vectors formed by the values of induced currents for each of the nodes of each element ($J^{i}=\sigma^{i}\left(\frac{\partial A_{x}^{i}}{\partial t}+\frac{\partial A_{y}^{i}}{\partial t}+\frac{\partial A_{y}^{i}}{\partial t}\right)$ and $P_{ij}^{\prime }=-\mu_{o}\sigma_{i}P_{ij}$).

#### 4.2.3. Solution of the Darwin Model BVP

It can be seen from Equations (35a) and (35b) or their versions considering the Coulomb gauge that they can be integrated using the procedure explained in the two previous sections by adding a new source term to Equations (34a) and (34b) and considering the change of potential due to the presence of displacement currents in the second equation. Considering the Coulomb gauge and that the electric permittivity (ϵ) is time-independent, Equations (35a) and (35b) can be expressed as:

(101a) $$\begin{array}{cc} \; & -\frac{1}{\mu_{o}}{\vec{\nabla }}^{2}\vec{A}+\sigma \left(\frac{\partial A}{\partial t}+\vec{\nabla }\phi \right)+\epsilon \vec{\nabla }\frac{\partial \phi }{\partial t}={\vec{J}}_{s} \end{array}$$

(101b) $$\begin{array}{cc} \; & \vec{\nabla }\;\cdot \;\left(\sigma \left(\frac{\partial \vec{A}}{\partial t}+\vec{\nabla }\phi \right)\right)+\vec{\nabla }\;\cdot \;\left(\epsilon \vec{\nabla }\frac{\partial \phi }{\partial t}\right)=\nabla \;\cdot \;{\vec{J}}_{s} \end{array}$$

where the new source term for the first equation is $\epsilon \vec{\nabla }\frac{\partial \phi }{\partial t}$, and the new scalar potential term in the second equation is $\vec{\nabla }\;\cdot \;\epsilon \vec{\nabla }\frac{\partial \phi }{\partial t}$. Equations (101a) and (101b) can be further modified by approximating the scalar potential using a finite difference scheme:

(102a) $$\begin{array}{cc} \; & -\frac{1}{\mu_{o}}{\vec{\nabla }}^{2}\vec{A}+\sigma \left(\frac{\partial A}{\partial t}+\vec{\nabla }\phi^{t}\right)+\frac{\epsilon }{\Delta t}\vec{\nabla }\phi^{t}-\frac{\epsilon }{\Delta t}\vec{\nabla }\phi^{t-\Delta t}={\vec{J}}_{s} \end{array}$$

(102b) $$\begin{array}{cc} \; & \vec{\nabla }\;\cdot \;\left(\sigma \left(\frac{\partial \vec{A}}{\partial t}+\vec{\nabla }\phi \right)\right)+\vec{\nabla }\;\cdot \;\left(\frac{\epsilon }{\Delta t}\vec{\nabla }\phi^{t}\right)-\vec{\nabla }\;\cdot \;\left(\frac{\epsilon }{\Delta t}\vec{\nabla }\phi^{t-\Delta t}\right)=\nabla \;\cdot \;{\vec{J}}_{s} \end{array}$$

The term $\vec{J_{dis}}=\frac{\epsilon }{\Delta t}\vec{\nabla }\phi^{t-\Delta t}$ is a displacement current density that comes from the potential induced in the previous time instant. The term $-\sigma \frac{\partial \vec{A}}{\partial t}$ is the conductive current density (${\vec{J}}_{ind}$), which is magnetically induced. Therefore, these equations can be rearranged as:

(103a) $$\begin{array}{cc} \; & \sigma \frac{\partial A}{\partial t}-\frac{1}{\mu_{o}}{\vec{\nabla }}^{2}\vec{A}+\left(\sigma +\frac{\epsilon }{\Delta t}\right)\vec{\nabla }\phi^{t}={\vec{J}}_{s}+{\vec{J}}_{dis} \end{array}$$

(103b) $$\begin{array}{cc} \; & \vec{\nabla }\;\cdot \;\left(\sigma +\frac{\epsilon }{\Delta t}\right)\vec{\nabla }\phi^{t}=\vec{\nabla }\;\cdot \;\left({\vec{J}}_{s}+{\vec{J}}_{dis}+{\vec{J}}_{ind}\right) \end{array}$$

which essentially have the same form as the A-Φ formulation, with an additional current source. Therefore, this equation set can be solved using the finite difference method as described in Section 3.1.

#### 4.2.4. Solution of the Darwin Model BVP Using FEM

Houssein Taha [60] proposed the solution of the Darwin model directly using Equations (35a) and (35b) applied to electric machines. However, working with the numerical curl of vectors usually requires a high computational cost if applied to a complex geometry mesh such as the brain cortex. Based on the modification of the equations described in the previous section, the Darwin model for TMS applications can be reduced to a type of Φ-V formulation. Therefore, comparing Equations (34a) and (34b) and their FEM numerical solution (Equation (100)) with Equation (103), the FEM formulation for the Darwin model can be expressed as:

(104) $$\left(\begin{array}{cc} \left[P+T\right] & R^{\prime }\\ 0 & P^{\prime \prime } \end{array}\right)\left(\begin{array}{c} A^{t}\\ \Phi \end{array}\right)=\left(\begin{array}{c} -TA^{t-\Delta t}-S^{\prime }\\ -QJ^{\prime } \end{array}\right)$$

where P, T and Q are the same as in the Φ-V formulation solution, and $R_{ij}^{\prime }=\beta_{\sigma \epsilon }^{i}\int_{\Omega }\frac{\partial N_{i}}{\partial x}N_{j}dV$, where $\beta_{\sigma \epsilon }^{i}=\left(\sigma^{i}+\frac{\epsilon^{i}}{\Delta t}\right)$, is constant for the nodes of each element. $P_{ij}^{\prime \prime }=-\mu_{o}\beta_{\sigma \epsilon }^{i}P_{ij}$, $S_{v}^{\prime }=F_{v}^{\prime }+B_{v}$ for v={x,y,z}, with $F^{\prime }=-\left(J_{s}+J_{dis}\right)\int_{\Omega }N_{i}dV$ and $J^{\prime }=\left(J_{s}+J_{dis}\right)$.

### 4.3. Analytical and Semianalytical Solutions

It is clear that it is very difficult—if not impossible—to obtain an analytic solution of the TMS field formulations on realistic brain cortex geometries. However, it is possible to obtain analytic solutions considering simplified geometric models of the excitation coil and the brain. The advantage of these solutions is that they serve as a calibration reference for numerical simulations. Esselle et al. [49] computed the electric field produced by an arbitrarily shaped coil above half-space tissue with a planar interface with the air. The formulation corresponds to the Laplace MQS-ϕ formulation:

(105a) $$\begin{array}{cc} \; & \nabla^{2}\vec{A}=-\mu_{o}J_{s},/\left\{\vec{A}\subset \Omega ,\nabla \times {\vec{A}}_{\Gamma }=0\right\} \end{array}$$

(105b) $$\begin{array}{cc} \; & \nabla^{2}\phi =0,/\left\{\phi \subset \Omega_{b},n_{1}\;\cdot \;{\vec{J_{1}}}_{\Gamma }=n_{2}\;\cdot \;{\vec{J_{2}}}_{\Gamma }\right\} \end{array}$$

The solution of the magnetic vector potential generated by a coil formed by *N* thin wires carrying a current (*I*) is:

(106) $$\vec{A}\left(r_{o}\right)=\frac{\mu_{o}NI}{4\pi }\oint_{\Sigma_{coil}}^{}\frac{1}{\left|r-r_{o}\right|}d\vec{l}$$

The primary electric field can be computed from the magnetic potential vector; therefore:

(107) $$d\vec{E_{p}}=-\frac{\mu_{o}N\left(dI/dt\right)}{4\pi \left|r-r_{o}\right|}d\vec{l}$$

The secondary electric field (${\vec{E}}_{s}$) of a coil element ($d\vec{l}$) is computed from the scalar potential as:

(108) $$d\vec{E_{s}}=-\vec{\nabla }\phi =-\frac{\partial \phi }{\partial x}i-\frac{\partial \phi }{\partial y}j-\frac{\partial \phi }{\partial z}k$$

Consequently, the total electric field of the coil element ($d\vec{l}$) is given as:

(109) $$d\vec{E}=d\vec{E_{p}}+d\vec{E_{s}}=\left(dE_{p}^{x}-\frac{\partial \phi }{\partial x}\right)i+\left(dE_{p}^{y}-\frac{\partial \phi }{\partial y}\right)j+\left(dE_{p}^{z}-\frac{\partial \phi }{\partial z}\right)k$$

Considering the condition of the continuity of the current in the interfaces, since the electric conductivity of the air is zero, then the current in the air–tissue interface produced by the $d\vec{l}$ element of the coil perpendicular to the tissue interface is in the z direction; therefore:

(110) $$\vec{J_{b}}=\sigma_{b}d\vec{E}=\sigma_{b}d\vec{E^{z}}=0$$

or

(111) $$d\vec{E^{z}}=0$$

Therefore, it is only necessary to compute $dE^{x}$ and $dE^{y}$. Furthermore, the boundary condition Equation (111) can be used to compute these components. From the boundary condition ($d\vec{E_{z}}=0$), it is deduced that at the interface is z=0:

(112) $$-\frac{\mu_{o}N\left(dI/dt\right)}{4\pi \left|r-r_{o}\right|}dz-\frac{\partial \phi }{\partial z}=0$$

A general solution of the Laplace Equation (105b) can also be expressed as the Bessel integral:

(113) $$\phi =\int_{0}^{\infty }U\left(\lambda \right)e^{\lambda z}J_{o}\left(\lambda \rho \right)d\lambda$$

where $\rho =\sqrt{x^{2}+y^{2}}$, and $J_{o}$ is the Bessel function of the first kind. Replacing Equation (113) in Equation (112) and also using the expansion of $\frac{1}{\left|r-r_{o}\right|}$ as a function of the Bessel function ($J_{o}$), the value of ϕ is obtained as:

(114) $$\phi =-\frac{\mu_{o}N\left(dI/dt\right)}{4\pi }dl_{z}\int_{0}^{\infty }\frac{1}{\lambda }e^{-\lambda (z_{o}-z)}J_{o}\left(\lambda \rho \right)d\lambda$$

This expression is replaced in Equation (109) to obtain components $dE^{x}$ and $dE^{y}$, which are given by:

(115) $$dE^{x}=dE_{p}^{x}-\frac{\partial \phi }{\partial x}=-\frac{\mu_{o}N\left(dI/dt\right)}{4\pi }\left[\frac{dl_{x}}{R}+\frac{xdl_{z}}{\rho^{2}}\left(1-\frac{z-z_{o}}{R}\right)\right]$$

and

(116) $$dE^{y}=dE_{p}^{y}-\frac{\partial \phi }{\partial y}=-\frac{\mu_{o}N\left(dI/dt\right)}{4\pi }\left[\frac{dl_{y}}{R}+\frac{ydl_{z}}{\rho^{2}}\left(1-\frac{z-z_{o}}{R}\right)\right]$$

The differential of the total electric field is obtained as $dE=dE^{x}i+dE^{y}j$.

Although the work by Esselle et al. [49] starts from the approximation that the tissue interface is plane, they also conclude that the result was independent of the tissue inhomogeneities inside the tissue half-plane if the conductivity of the inhomogeneous tissue changes perpendicular to the location of the interface.

A more precise geometrical model was used in the H. Eaton model [48], which proposed an analytical expression to compute the electric field and current density induced by a coil inside a homogeneous spherical coil. The author used the Laplace MSQ-V formulation as described in Section 3.3. Therefore, the solution of the magnetic potential vector is given by Equation (36). To analytically integrate this expression, the inverse of the distance term is expanded in terms of spherical harmonics:

(117) $$\frac{1}{\left|r-r_{o}\right|}=4\pi \sum_{l=0}^{\infty }\sum_{m=-l}^{l}\left[\frac{r^{l}}{\left(2l+1\right)r_{o}^{l+1}}\right]Y_{lm}^{*}\left(\theta_{o},\phi_{o}\right)Y_{lm}\left(\theta ,\phi \right),\;\;r_{o}>r$$

where $Y_{lm}\left(\theta ,\phi \right)$ represents the spherical harmonic functions, and * indicates a complex conjugate. After replacing this expression in Equation (36), the value of the magnetic potential vector is:

(118) $$\vec{A}=\mu_{o}I\sum_{l=0}^{\infty }\sum_{m=-l}^{l}\frac{r^{l}}{\left(2l+1\right)}{\vec{C}}_{lm}Y_{lm}\left(\theta ,\phi \right)$$

where

(119) $${\vec{C}}_{lm}=\oint_{\Omega_{coil}}\frac{Y_{lm}^{*}\left(\theta_{o},\phi_{o}\right)}{r_{o}^{l+1}}d{\vec{l}}_{o}$$

The values of the coefficient (${\vec{C}}_{lm}$) are obtained by dividing the domain ($\Omega_{b}$) into rectangular components such that:

(120) $${\vec{C}}_{lm}=C_{lm}^{x}i+C_{lm}^{y}j+C_{lm}^{z}k$$

Moreover, ϕ is obtained after integrating the Laplace equation ($\nabla^{2}\phi =0$), which is the other equation of the the Laplace MQS-ϕ formulation, with boundary conditions given by the current continuity Equation (32d) and the electric potential outside the brain (sphere of radius (*R*)), vanishing (ϕ=0) for r→∞:

(121) $$\phi =\sum_{l=0}^{\infty }\sum_{m=-l}^{l}\left(F_{lm}+G_{lm}\right)Y_{lm}\left(\theta ,\phi \right)$$

where

(122) $$G_{lm}=F_{lm}R^{2l+1}$$

(123) $$\begin{array}{c} F_{lm}=-\frac{j\omega \mu_{o}I\left[\sigma +j\omega (\epsilon -\epsilon_{o})\right]}{l\left(\sigma +j\omega \epsilon \right)+j\omega \epsilon_{o}\left(l+1\right)}\left(-\alpha_{lm}D_{l-1,m-1}+\beta_{lm}E_{l-1,m+1}+\gamma_{lm}C_{lm}^{z}\right) \end{array}$$

with $\alpha_{lm}=\sqrt{\frac{\left(l+m-1\right)\left(l+m\right)}{\left(2l+1\right)\left(2l-1\right)}}$, $\beta_{lm}=\sqrt{\frac{\left(l-m-1\right)\left(l-m\right)}{\left(2l+1\right)\left(2l-1\right)}}$, $\gamma_{lm}=\sqrt{\frac{\left(l-m\right)\left(l+m\right)}{\left(2l+1\right)\left(2l-1\right)}}$, $D_{lm}=\frac{C_{lm}^{x}-jC_{lm}^{y}}{2}$ and $E_{lm}=\frac{C_{lm}^{x}+jC_{lm}^{y}}{2}$ for l>0 and $F_{oo}=0$. Until this point, it is possible to obtain a semianalytical solution of the formulation by numerically integrating Equation (119) and computing $\vec{A}$ and ϕ using Equations (118) and (121), respectively, and the electric field using Equation (6). However, the authors took a step forward by replacing the analytic expressions with their corresponding spherical harmonic functions and obtaining analytical expressions for the electric field component in spherical coordinates, as described in [48].

## 5. Implementation of Numerical Solutions

### Review of Solutions and Implementations Presented in the Literature

Table 3 provides a comprehensive overview of the predominant BVPs employed in the simulation of TMS fields. Alongside these BVPs, we present the corresponding Simulation Tools and their respective authors who have utilized them. The term "Direct implementation" pertains to cases where the authors have custom-built their simulation codes using programming languages, tailoring them to the specific requirements of their studies. On the other hand, "Commercial general-purpose software" refers to computer simulation programs, frequently employing the finite element method, capable of simulating various multiphysics problems represented by partial differential equations and boundary conditions.

## 6. Overview of Some of the Main TMS Simulation Results

The effectiveness and applications of TMS depend on several parameters of the coil-brain system, as mentioned in the Introduction. Some analyses using simulation methods have revealed how these parameters influence the electric field acting on the nerves. The use of different formulations, brain models and levels of numerical method precision can also influence the accuracy of estimating the field. A brief overview of some of these results is presented below.

Although it is difficult to establish a level of priority of elements that influence the electric field induced in the brain by TMS and the computation of the actual field distribution in the brain, there seems to be a consensus among works [16,18,20,21,22,23,24] related to the simulation of the electric field induced by TMS, which analyzed the influence of the coil parameters (geometry, positioning, current waveform, etc.). Their main conclusion is that the adequate selection of these parameters is crucial for inducing the value of the electric field. In other words, the excitation parameters are the most fundamental elements for achieving the necessary value and distribution of the electric field in the brain, and the smallest variation of these parameters could have a significant impact on the excitation field.

Research conducted by Mills [20] demonstrated that the utilization of single coils for the purpose of stimulating long nerves is not advantageous. This is attributed to the presence of “hot spots” within the induced electric field generated by such coils, which possess both positive and negative fields. These hot spots can elicit contradictory effects on the nerve. Additionally, through the simulation of various coil geometries and configurations, Deng [17] established that figure-8-type coils exhibit greater focal properties compared than circular-type coils. None of the simulated coil configurations surpassed the figure-8-type coils in terms of achieving a tradeoff between depth of penetration and focal precision. Furthermore, Deng’s research [17] indicated that an increase in the number of coil turns (N) amplifies coil inductance and peak voltage while reducing the necessary peak current. Theoretically, augmenting N enhances the accuracy of field replication, although this is constrained by the coil’s dimensional requirements.

The impact of TMS is heavily influenced by the parameters employed for excitation. Specifically, the electric field distribution, which crucial for effective stimulation, may not always achieve the desired level of focus due to the configuration of the excitation coil in TMS. Consequently, achieving highly localized stimulation through the appropriate selection and arrangement of coils can prove challenging for specific applications. In this regard, a novel emerging technique called transcranial ultrasound stimulation (TUS) [101,102] offers several advantages, including the ability to provide highly focused excitation using an ultrasonic transducer. Moreover, the utilization of ultrasonic waves allows for deeper penetration of the stimulus into the brain, enabling access to central brain regions. Nevertheless, despite being considered a safe technique, further research is necessary to establish the long-term safety and potential risks associated with TUS. Additionally, the use of coupling gel is required for excitation in TUS. Furthermore, the indirect mechanism by which ultrasound stimulates neural activity through mechanical oscillations is more intricate compared to the direct electrical stimulation employed by TMS.

In reference to the precision of the estimation of the induced electric field, the accuracy of the coil and current parameters used in the model is very important, as well as an adequate formulation and BVP [20,23,24]. In order of significance, the correct assignment of electric conductivity to each point in the brain model [30,31,32,33,34] and the selection of an appropriate numerical method for the simulations [73,90] are the subsequent key factors to be considered. Lastly, there are the details of brain model geometry, provided the use of a basic brain model including most of the fundamental parts (gyrus and sulcus) of the brain is used, although there is no clear consensus about the importance of the later. In this review, the effect of the shape and length of neurons and nerves, [103,104,105] which is also of fundamental importance, is not considered.

Petrov’s study [58] reported that in the specific region where neurostimulation is typically performed, spiral wire coil models exhibited higher precision (RE < 5%) in predicting the actual induced field compared to a single circular wire coil model, which deviated from field measurements by up to 10% RE. These findings emphasize the necessity of employing a spiral winding-turns model to accurately approximate the induced field of a typical TMS coil [58]. Moreover, the utilization of realistic magnetic resonance imaging (MRI)-derived head models demonstrated that differences in tissue conductivities had a negligible impact on the magnitudes of the electric field (E-field). However, the position and orientation of the coil, as well as the size of the brain, exerted significant effects on the magnitude of the E-field [24]. Notably, substantial deviations in E-field magnitude were observed with respect to coil placement when considering the evaluation of effects over a 2 cm range in each direction of a two-dimensional plane of the TMS coil [24]. This estimation may be regarded as conservative when assessing the disparity between the scalp location and the intended cortical target. The results highlight the extensive cortical area that may be affected when accounting for this level of positioning uncertainty, encompassing a significant portion of the dorsolateral prefrontal cortex [24]. Certainly, it is feasible to reduce this uncertainty within an individual patient by developing reliable coil placement procedures combined with neuronavigation techniques [24,58].

On the other hand, in a recent study [83], it was discovered that the standard deviations in maximum electric field values resulting from uncertainties in tissue conductivities accounted for approximately 5% of the mean value in TMS. Tissue conductivity emerged as the primary factor determining the magnitudes of current density when the displacement current was insignificant [83]. Conversely, with the presence of displacement currents, the permittivity became the principal determinant of current density magnitude. Furthermore, the study revealed that the existence of displacement currents could potentially increase the maximum cortical current density by an order of magnitude, assuming the extreme permittivity values reported by certain researchers prove to be accurate [83]. Notably, all solutions exhibited currents that were perpendicular to the cortical interface, challenging models that propose preferential stimulation of tangentially oriented neurons based on the assumption that fields normal to the interface are negligible. Consequently, these models warrant re-examination [83].

The impact of brain models on simulation accuracy remains a contentious topic. Salvador et al. [96] demonstrated that the most consistent correspondence between the estimated electric field and motor activation was observed in realistic brain models, specifically in the crown region of the precentral gyrus. Conversely, the use of simplified head models, such as spherical head models, yielded spatially nonspecific findings [96]. This study also proposed that the maximum electric field strength serves as the optimal parameter for exploring the applicability of field calculations in quantitative dosing [62,96]. However, Janssen et al. [89] showed that in a highly realistic head model, variations in sulcus width (up to 3 mm) did not result in significant differences in the calculated electric field values for most brain areas. Thus, for a global estimation of the electric field, an accurate representation of sulci is of limited importance [89]. Nevertheless, considerable overestimation of sulcus width led to an overestimation of local field strength in locations distant from the cortical hot spot. In contrast, Saturnino et al. [85] demonstrated that accurate anatomical representation of tissue boundaries, including sulci, significantly improved the numerical accuracy of TMS field estimation in full-head models and sulcus models. Specifically, the sulcus model highlighted the importance of higher mesh density around highly curved regions of the gray matter/cerebrospinal fluid boundary, where electric potential undergoes rapid changes. This approach helped prevent local simulation errors and emphasized that the under-representation of sulci could lead to substantial errors in the electric field [85]. Moreover, modifications to cortical geometry were shown to disrupt stimulating fields, potentially impairing cortex targeting in non-normal populations [83].

Regarding numerical methods employed in simulations, it was found that the finite difference method (FDM) requires high spatial resolution in regular grids or hexahedral meshes to achieve detail comparable to that of tetrahedral meshes. However, this results in a large number of elements, necessitating lengthy computation times and significant computational resources. Therefore, the finite element method (FEM) based on tetrahedral meshes was suggested as better-suited for these calculations [90].

## 7. Conclusions

Herein, we present a survey of the most used formulations, boundary value problems and implementations for the numerical computation of TMS fields. These aspects were analyzed, showing theirs limitations and advantages. The deduction of the formulations from the Maxwell equations and the numerical solution of the corresponding BVP were described in detail. We also induced some numerical solutions not found in the consulted literature, such as finite difference for quasistatic magnetic A-ϕ and Darwin models and finite element for quasistatic magnetic A-ϕ and Darwin models using the Galerkin method, as well as their implementation of boundary conditions applied to TMS fields. The aim of the present work was to serve as a guide for reproduction and future implementation of TMS simulations.

Several of the main results deduced from the TMS fields simulations emphasize the importance of the stimulation parameters, in particular, the coil parameters and arrangement, followed by the precision of the specification of the electrical properties of the tissue and details of the brain geometry model. Our literature analysis highlights that the most used approach is the Poisson MQS-ϕ formulation applied to a model consisting a figure-8-type coil and a brain model. The advantage of this method is that it does not require large computational resources and enables consideration of the effects of the formation of electric charges in the inner interfaces of the brain. In this case, computational resources can also be redirected to increase the detail of the brain model geometry. Laplace MQS approximation requires significantly fewer computational resources. However, apart from its limitation in considering the charges formed at the inner interfaces, it is practically limited in terms of setting the boundary condition in the complex brain-to-air interface, which usually requires an advance graphical interface. The use of commercial software is common practice in the reviewed works. Nevertheless, the use of software has its drawbacks, as it limits the flexibility of connection in the simulated electric field output with the input for neuron equations and models. The use of a direct implementation could solve these limitations, but there is still a considerable range of code implementations and source code styles, which limits the widening of their use. The recent development of TMS custom platforms such as SimNIBS represents a step forward in solving these drawbacks, although the tendency of the development of graphical interfaces that should be mastered could affect the effectiveness of such platforms. In that sense, it may be more useful to work towards the development of flexible toolboxes and libraries for TMS simulations.

## Figures and Tables

**Table 1:** Domains, their boundaries, and electric and magnetic properties.

Domain	Boundary	Applicable Properties	Description
Ω	Γ	$\sigma \neq 0,\epsilon_{r}\neq 1,\mu_{r}=1$	System domain that includes all other domains: $\Omega \;=\;\Omega_{a}\cup \Omega_{c}\cup \Omega_{b}$. Its boundary is limited by the air exterior boundary: $\Gamma \cap \Gamma_{a}=\Gamma$
$\Omega_{a}$	$\Gamma_{a}$	$\sigma =0,\epsilon_{r}=1,\mu_{r}=1$	Air
$\Omega_{c}$	$\Sigma_{c}$ (integration path)	$\sigma =\left\{0,\sigma_{cupper}\right\},\epsilon_{r}=1,\mu_{r}=1$	Coil
$\Omega_{b}$	$\Gamma_{b}$	$\sigma \neq 0,\epsilon_{r}\neq 1,\mu_{r}=1$	The brain, composed of several brain tissues ($\Omega_{b}\;=\;{⋃}_{i=0}^{n}\Omega_{b}^{i}$); its boundary is limited by the air ($\Gamma_{a}\cap \Gamma_{b}=\Gamma_{b}$ )
$\Omega_{b}^{i}$	$\Gamma_{b}^{i}$	$\sigma \neq 0,\epsilon_{r}\neq 1,\mu_{r}=1$	Brain tissues. Some of these domains are considered to, partially share boundaries ($\Gamma_{b}^{i}\cap {⋃}_{j=0}^{n}\Gamma_{b}^{j}\;\neq \;O,\forall i\neq j$)

**Table 2:** Studies of TMS numerical simulation-based MQS-ϕ boundary value problems.

BVP	Model	Publications
MQS only $E_{p}$	Several coils and coil configurations in an air box	[21,50]
	A coil over a passive cable	[42]
	Figure-8 coil over a tissue planar interface	[44]
	A quasispherical volume conductor and a paired coil	[71]
	A neurocortical neuron model and a coil	[77]
$E_{p}$ + skin effect	A coil over an non-homogeneous volume conductor	[54]
Laplace MQS-ϕ	A coil over a cylindrical volume conductor	[43]
	Three types coils over a spherical volume conductor	[48]
	An arbitrarily shaped coil over a half-plane conductor	[49]
	A circular coil over a spherical conductor	[72,88,97]
	A coil over a half-plane tissue	[84]
Poisson MQS-ϕ	Figure-8 coil over a realistic brain model	[22,56,58,75,79,80,81,82,85,87,90,91,92,94]
	A circular coil over a parallelepiped volume conductor	[47,51,95]
	A coil over an approximate brain model	[52,93]
	Figure-8 coil over several brain models	[73]
	Figure-8 coil over a high-resolution brain model	[74,86]
	Uniform and realistic E-fields and a realistic brain model	[78]
MQS A-ϕ	An 8-shaped coil over a cortical sulcus	[39,98]
	A circular coil over a realist head model	[53]
	A custom coil over three concentric spheres	[76]
	Figure-8 coil over an approximate head model	[83]
MQS full Maxwell equations	Figure-8 coil over a brain approximate model	[62]

**Table 3:** TMS numerical simulation studies based on different BVPs.

Boundary Value Problem	Simulation Tool	Type	Publications
MQS only Ep	Matlab	Direct implementation	[21]
	Equations solved with VAX 750 and Fortran	Direct implementation	[42]
Laplace MQS-ϕ	FEM using version 4.7 of SCIRun: A Scientific Computing Problem Solving Environment (SCI), Utah, USA	Custom software	[58]
	FEM model using Matlab and the GetFEM++ library	Direct implementation	[75,89]
	Comsol multiphysics	Commercial general purpose software	[96]
Poisson MQS-ϕ	FEM custom-written Matlab and C++ routines together with Getfem++ functions	Direct implementation	[22]
	FEM software	Unknown	[52]
	Ansoft finite-element package	Commercial general-purpose software	[97]
	SimNIBS pipeline	Open-source software for TMS simulation	[56,94]
	SimBio software environment	Open-source software for TMS simulation	[74,81,82]
	SimNIBS	Open-source software for TMS simulation	[78,79,85,93]
	Matlab and C++	Direct implementation	[80,90,91]
	Matlab FDM	Direct implementation	[92]
	I-DEAS FEM package	Commercial general-purpose software	[95]
MQS A-ϕ	FEM implemented by Comsol Multiphysics	Commercial general-purpose software	[39]
	Eddy current solver from the commercial FEM package Maxwell3D from Ansoft	Commercial software	[53,83,86]
Full Maxwell equations	FEM implemented by Comsol Multiphysics	Commercial general-purpose software	[62]

## Data Availability

Not applicable.

## Abbreviations

**for Maxwell Equations**	
$\vec{E}$	Electric field
$\vec{D}$	Electric flux density
$\vec{H}$	Magnetic field
$\vec{B}$	Magnetic flux density
$\vec{A}$	Magnetic potential vector
ϕ	Electric scalar potential
ρ	Electric charge density
${\vec{J}}_{s}$	Vector of external (excitation) current density
${\vec{J}}_{ind}$	Vector of induced current density
${\vec{J}}_{disp}$	Vector of displacement current density
σ	Electric conductivity
$\varepsilon_{0}$	Electric permittivity of free space
ε	Material electric permittivity
$\mu_{0}$	Magnetic permeability of free space
μ	Material magnetic permeability
ω	Frequency of the excitation current
$j=\sqrt{-1}$	Complex number imaginary unit
**for Numerical Methods**	
$\phi_{i,j,k}$	Electric scalar potential
$\Phi^{e}$	Electric scalar potential vector of element *e*
Φ	Electric scalar potential vector for all elements in the domain Γ
$J_{i,j,k}^{k}$	Nodal current density with the component k={x,y,z}
$J^{e}$	Current density vector of the element *e*
*J*	Current density vector of all the elements in the domain Γ
$A_{i,j,k}^{k}$	Nodal magnetic potential of the component k={x,y,z}
$A^{e}$	Magnetic potential vector for the element *e*
$A_{k}$	Magnetic potential k-component for all elements in the domain Γ
